# Prevalence of risk factors for primary postpartum hemorrhage in a university hospital

**DOI:** 10.1590/0034-7167-2022-0134

**Published:** 2023-11-27

**Authors:** Thaís Betti, Helga Geremias Gouveia, Vanessa Aparecida Gasparin, Letícia Becker Vieira, Juliana Karine Rodrigues Strada, Júlia Fagherazzi

**Affiliations:** I Universidade Federal do Rio Grande do Sul. Porto Alegre, Rio Grande do Sul, Brazil

**Keywords:** Postpartum Hemorrhage, Postpartum Period, Risk Factors, Prevalence, Maternal Health, Hemorragia Posparto, Periodo Posparto, Factores de Riesgo, Prevalencia, Salud Materna, Hemorragia Pós-Parto, Período Pós-Parto, Fatores de Risco, Prevalência, Saúde Materna

## Abstract

**Objectives:**

to identify the risk factors associated with primary postpartum hemorrhage in a university hospital.

**Methods:**

a cross-sectional study was conducted with 277 postpartum women who received care during childbirth or cesarean section between June and August 2020. Data were collected using a pre-structured questionnaire administered 24 hours after delivery. Poisson Regression was employed to analyze the factors associated with postpartum hemorrhage.

**Results:**

postpartum hemorrhage was observed in 30% of the study sample. Shock Index and uterine distension were found to be statistically associated with postpartum hemorrhage. Postpartum women with a Shock Index ≥ 0.9 had a 61% higher prevalence of postpartum hemorrhage (PR=1.61, 95% CI: 1.07 - 2.43), while those with uterine distension had a 134% higher prevalence (PR=2.34, 95% CI: 1.63 - 3.36).

**Conclusions:**

recognizing these factors contributes to improvements in clinical practice, as they enable the prediction of their occurrence and call for appropriate management, thereby preventing unfavorable outcomes.

## RESUMO

**Objetivos:**

identificar os fatores de risco associados à hemorragia pós-parto primária em um hospital universitário.

**Métodos:**

estudo transversal realizado com 277 puérperas que receberam assistência durante o parto ou cesárea no período de junho a agosto de 2020. Os dados foram coletados por meio de um questionário previamente estruturado, aplicado após 24 horas do nascimento. Para a análise dos fatores associados à hemorragia pós-parto, utilizou-se a Regressão de Poisson.

**Resultados:**

a hemorragia pós-parto foi observada em 30% da amostra do estudo. O Índice de Choque e a distensão uterina foram estatisticamente associados à hemorragia pós-parto. Puérperas com Índice de Choque ≥ 0,9 apresentam uma prevalência 61% maior de hemorragia pós-parto (RP=1,61; IC 95%: 1,07 - 2,43) e com distensão uterina 134% (RP=2,34; IC 95%: 1,63 - 3,36).

**Conclusões:**

o reconhecimento desses fatores subsidia melhorias na prática clínica, visto que possibilitam a previsão de sua ocorrência e alertam para o manejo adequado, prevenindo desfechos indesejáveis.

## RESUMEN

**Objetivos:**

identificar los factores de riesgo asociados con la hemorragia posparto primaria en un hospital universitario.

**Métodos:**

se realizó un estudio transversal con 277 mujeres posparto que recibieron atención durante el parto o la cesárea entre junio y agosto de 2020. Los datos se recopilaron mediante un cuestionario preestructurado administrado 24 horas después del parto. Se empleó una Regresión de Poisson para analizar los factores asociados con la hemorragia posparto.

**Resultados:**

se observó hemorragia posparto en el 30% de la muestra del estudio. El índice de choque y la distensión uterina se encontraron estadísticamente asociados con la hemorragia posparto. Las mujeres posparto con un índice de choque ≥ 0,9 tenían una prevalencia de hemorragia posparto un 61% mayor (PR=1,61, IC del 95%: 1,07 - 2,43), mientras que aquellas con distensión uterina tenían una prevalencia un 134% mayor (PR=2,34, IC del 95%: 1,63 - 3,36).

**Conclusiones:**

reconocer estos factores contribuye a mejoras en la práctica clínica, ya que permiten predecir su ocurrencia y requieren una gestión adecuada, previniendo así resultados desfavorables.

## INTRODUCTION

Postpartum Hemorrhage (PPH) is an obstetric emergency and is recognized as one of the primary causes of maternal mortality in low-income countries, accounting for nearly a quarter of all maternal deaths worldwide^(1)^. Consequently, one of Brazil’s Sustainable Development Goals is to reduce global maternal mortality to a maximum of 30 deaths per 100,000 live births by 2030^(2)^.

PPH is defined as a blood loss of 500 ml or more in vaginal deliveries and 1000 ml or more in cesarean sections within the first 24 hours after childbirth, or any blood loss from the genital tract that can result in hemodynamic instability. It is considered massive when the volume of bleeding exceeds 2000 ml within the initial 24 hours following delivery, when a minimum transfusion of 1200 ml of packed red blood cells is required, or when it leads to a decrease in hemoglobin levels of ≥ 4g/dl or coagulation disorders^(3-4)^.

The Pan American Health Organization (PAHO) classifies PPH as primary when bleeding occurs within the first 24 hours after delivery and as secondary when it happens after 24 hours and up to six weeks postpartum^(4)^. The majority of PPH-related deaths occur within the initial 24 hours after childbirth, which can be prevented by administering prophylactic uterotonics during the third stage of labor and providing timely and appropriate care^(1)^.

Evidence suggests that despite the majority of deliveries in Brazil taking place in healthcare institutions equipped to handle PPH cases and having public policies for postpartum care, the country still faces significant challenges in reducing maternal deaths caused by PPH. This highlights the need to improve management protocols and implement actions that ensure maternal well-being during the postpartum period^(5)^.

When analyzing global cases of maternal deaths due to PPH, it becomes apparent that there are gaps in access to quality healthcare services for users, obstetric interventions for hemorrhage, and organizational and structural issues in healthcare facilities. These factors, either individually or collectively, contribute to delays in appropriate management of postpartum bleeding. On the other hand, the involvement of trained professionals in the management of obstetric emergencies, early diagnosis, prevention, and treatment of PPH is crucial in reducing maternal morbidity and mortality^(4)^.

The primary objective in managing PPH is to achieve control over bleeding and ensure that the patient recovers from shock within 60 minutes of diagnosis, known as the “Golden Hour,” as survival rates are inversely proportional to the time taken for recovery from this condition^(4)^.

While discussions on diagnosing and treating PPH exist, there remains a scarcity of studies exploring risk factors specific to the Brazilian context. Recognized factors include preeclampsia, a history of previous hemorrhage during deliveries, multiple pregnancies, previous cesarean section scars, multiparity, prolonged third stage of labor, episiotomies, operative delivery, and fetal macrosomia^(3)^. The presence of these factors can initiate a cascade of events that may result in maternal mortality, with an analysis of data from 1997 to 2009 revealing over 22,000 deaths, 14.2% of which were associated with hemorrhage^(5)^.

Despite up-to-date international recommendations, PPH rates remain high, making it the leading cause of maternal death worldwide^(1)^. Therefore, research focused on the factors associated with PPH continues to hold significance, offering potential novel perspectives that can be further explored, particularly within institutions specialized in high-risk pregnancy and postpartum care.

Considering the crucial importance of early identification of postpartum women at risk of PPH and the potential for improved care quality to contribute to reducing maternal mortality, the justification for undertaking this study becomes apparent. Recent research has identified PPH as a major contributor to severe acute maternal morbidity^(6)^.

The findings of this study can serve as a basis for proposing reevaluation and enhancements in care practices, specifically aimed at preventing PPH. Furthermore, they can aid in identifying postpartum women at a higher risk of developing this complication, thus contributing to improved management strategies.

## OBJECTIVES

To identify the risk factors associated with primary postpartum hemorrhage in a university hospital.

## METHODS

### Ethical aspects

This project was approved by the Research Ethics Committee of the Hospital de Clínicas de Porto Alegre. The development of this research followed all ethical norms and guidelines as established in Resolution of the National Health Council No. 466/12 regarding research involving human subjects.

### Study Design, Period, and Location

This is a quantitative, analytical, cross-sectional study conducted in accordance with the Strengthening the Reporting of Observational Studies in Epidemiology (STROBE) guidelines. Data collection took place from June 20, 2020, to August 25, 2020. The data were collected from medical records obtained from the Obstetric Center Unit (OCU) and Obstetric Inpatient Unit (OIU) of a university hospital in southern Brazil.

### Sample; Inclusion and Exclusion Criteria

For the sample size calculation of this research, a prevalence of 16% for PPH was estimated, based on a previous study that estimated the prevalence of this complication. Considering a margin of error of 5 percentage points and a confidence level of 95%, the minimum sample size of 277 patients was determined. The statistical program WINPEPI version 11.65 was used to perform the sample size calculation.

The sample selection was done by convenience. Medical records that met the established inclusion and exclusion criteria were selected. The study included the medical records of postpartum women admitted to the Obstetric Inpatient Unit (OIU) and Obstetric Care Unit (OCU), regardless of gestational age and mode of delivery, with at least 24 hours postpartum, and containing all the necessary information for data collection.

Patients admitted through private healthcare providers (insurance) or self-financing (private) were excluded.

### Study Protocol

The outcome studied was the diagnosis of PPH, defined in conjunction with increased bleeding records and/or records of care interventions for PPH control, based on the institutional care protocol.

The independent variables encompassed sociodemographic data, obstetric history, current obstetric data, information about labor and delivery, and antepartum and intrapartum risk factors for PPH.

The data collection instrument was created by the researcher and structured based on the study variables. To verify the suitability of the data collection instrument, a pilot study was conducted, applying the instrument to 10 medical records.

Access to the information system was performed remotely, after authorization from the institution, due to the COVID-19 pandemic. After accessing the medical records, the data of interest for the research were collected and entered into the instrument created for this purpose.

### Analysis and Statistical Analysis

The collected data were organized into a database. Statistical analysis was conducted using the Statistical Package for the Social Sciences (SPSS) software, version 21.0. Quantitative variables were summarized using mean and standard deviation, while categorical variables were presented as absolute and relative frequencies.

To compare proportions between groups, Pearson’s chi-square test or Fisher’s exact test was employed. To account for confounding factors, a multivariate Poisson Regression model was utilized. All variables with a p-value <0.20 in the bivariate analysis were included in the multivariate model. A significance level of 5% (p≤0.05) was adopted to determine statistical significance.

## RESULTS

Among the 277 study participants, the majority were in the 24 to 34 age range, identified as white, and had completed high school. In relation to the primary outcome of interest, 30% (n=83) of the postpartum women experienced primary PPH, as determined by the documented management and analysis. Table 1 presents the variables associated with the characterization of the postpartum women included in the study.

**Table 1:** Bivariate analysis of postpartum women characteristics in the total sample (N=277) and according to Postpartum Hemorrhage, Porto Alegre, Rio Grande do Sul, Brazil, 2020

Variables	Total sample (N=277; 100%)	With PPH^*^ (n=83; 30%)	Without PPH^*^ (n=194; 70%)	*p* value
Age Range				0.630
14 to 19 years	34 (12.3)	11 (13.3)	23 (11.9)	
20 to 34 years	201 (72.6)	62 (74.7)	139 (71.6)	
35 years or older	42 (15.2)	10 (12.0)	32 (16.5)	
Race				0.957
White	206 (74.4)	62 (74.7)	144 (74.2)	
Black	49 (17.7)	15 (18.1)	34 (17.5)	
Mixed race	22 (7.9)	6 (7.2)	16 (8.2)	
Education Level				0.182
Incomplete elementary education	53 (19.1)	12 (14.5)	41 (21.1)	
Complete elementary education	38 (13.7)	13 (15.7)	25 (12.9)	
Incomplete secondary education	44 (15.9)	13 (15.7)	31 (16.0)	
Complete secondary education	110 (39.7)	39 (47.0)	71 (36.6)	
Incomplete higher education	16 (5.8)	5 (6.0)	11 (5.7)	
Complete higher education	16 (5.8)	1 (1.2)	15 (7.7)	
Primiparous (first-time mother)	101 (36.5)	33 (39.8)	68 (35.1)	0.542
Multiparous (had previous pregnancies)	176 (63.5)	50 (60.2)	126 (64.9)	0.542
Previous Delivery	135 (48.7)	41 (49.4)	94 (48.5)	0.990
Previous cesarean section	43 (15.5)	8 (9.6)	35 (18.0)	0.112
Previous abortion	50 (18.1)	15 (18.1)	35 (18.0)	1.000
Complications in Previous Pregnancies	45/176 (25.6)	16/50 (32.0)	29/126 (23.0)	0.298

* *Primary Postpartum Hemorrhage.*

Among the 176 multiparous participants included in the study, 45 experienced complications in previous pregnancies, representing 33.3% (n=15) with pre-eclampsia, 17.7% (n=8) with PPH, and 15.5% (n=7) who underwent curettage.

Table 2 presents the results of the bivariate analysis, examining the variables associated with the occurrence of PPH. Vaginal delivery (p=0.047), intravenous (IV) administration of oxytocin in the postpartum period (p<0.001), Shock Index (SI) ≥ 0.9 (p=0.028), and curettage (p=0.046) demonstrated a significant association with the outcome.

**Table 2:** Bivariate analysis for current pregnancy and delivery concerning the occurrence of Postpartum Hemorrhage, Porto Alegre, Rio Grande do Sul, Brazil, 2020

Variables	Total sample (N=277; 100%)	With PPH^*^ (n=83; 30%)	Without PPH^*^ (n=194; 70%)	*p* value
Twin pregnancy	5 (1.8)	1 (1.2)	4 (2.1)	1.000
Received prenatal care	264 (96.7)	80 (97.6)	184 (96.3)	0.728
Categorization of the number of prenatal visits				0.653
<6 visits	42 (15.9)	11 (13.8)	31 (16.8)	
≥6 visits	222 (84.1)	69 (86.3)	153 (83.2)	
Experienced complications during pregnancy	185 (66.8)	51 (61.4)	134 (69.1)	0.273
Delivery mode				0.047
Vaginal	192 (69.3)	65 (78.3)	127 (65.5)	
Cesarean	85 (30.7)	18 (21.7)	67 (34.5)	
Use of forceps	15/192 (7.8)	3/65 (4.6)	12/127 (9.4)	0.370
Laceration	83/192 (43.2)	27/65 (41.5)	56/127 (44.1)	0.854
Degree of laceration^†^				0.890
1st degree	49/81 (60.5)	16/27 (59.3)	33/54 (61.1)	
2nd degree	26/81 (32.1)	9/27 (33.3)	17/54 (31.5)	
3rd degree	5/81 (6.2)	2/27 (7.4)	3/54 (5.6)	
4th degree	1/81 (1.2)	0/27 (0.0)	1/54 (1.9)	
Suturing of the laceration	76/83 (91.6)	25/27 (92.6)	51/56 (91.1)	1.000
Episiotomy	57/192 (29.7)	18/65 (27.7)	39/127 (30.7)	0.790
Intravenous use of oxytocin during labor	155 (56.0)	48 (57.8)	107 (55.2)	0.780
Shock Index				0.028
< 0.9	238 (85.9)	65 (78.3)	173 (89.2)	
≥ 0.9	39 (14.1)	18 (21.7)	21 (10.8)	
Initiated labor	227 (81.9)	74 (89.2)	153 (78.9)	0.062

* *Primary Postpartum Hemorrhage; †In two cases, the degree of laceration was not recorded.*

In the immediate postpartum period, oxytocin is a medication used for preventing PPH. Among the 277 postpartum women, 235 received intravenous oxytocin, and out of those, 82 experienced PPH. Among women who had a vaginal delivery, five underwent retained placenta and curettage. Intramuscular oxytocin was administered to 164 participants (59.2%), and 182 of them (65.7%) had a prescription for this medication after delivery. Among the postpartum women diagnosed with PPH (n=83), it was found that 66.3% of them received both intramuscular and intravenous oxytocin in the postpartum period.

Regarding complications in the current pregnancy, among the 277 study participants, the following were observed: 20.9% had urinary tract infections, 18.8% had diabetes mellitus, 10.5% tested positive for Streptococcus B, and 10.1% had elevated blood pressure levels. There was no statistically significant association between the types of complications in the current pregnancy and the occurrence of PPH.

Regarding the risk factors for PPH, a significant association was found between uterine distention and elevated blood pressure levels during pregnancy, as demonstrated in Table 3.

**Table 3:** Bivariate analysis of antepartum and intrapartum risk factors for Postpartum Hemorrhage, Porto Alegre, Rio Grande do Sul, Brazil, 2020

Variables	Total sample (N=277; 100%)	With PPH^*^ (n=83; 30%)	Without PPH^*^ (n=194; 70%)	*p* value
Antepartum risk factors				
Uterine distensión	27 (9.7)	15 (18.1)	12 (6.2)	0.005
Coagulation disorder	2 (0.7)	1 (1.2)	1 (1.5)	0.510
Anticoagulant use	7 (2.5)	2 (2.4)	5 (2.6)	1.000
Multiple pregnancies	13 (4.7)	5 (6.0)	8 (4.1)	0.539
Elevated blood pressure levels during pregnancy^†^	37 (13.4)	5 (6.0)	32 (16.5)	0.031
Anemia during pregnancy	34/183 (18.6)	6/49 (12.2)	28/134 (20.9)	0.264
First childbirth after the age of 40	1 (14.3)	0 (0.0)	1 (14.3)	
Intrapartum risk factors				
Tachysystole of labor^‡^	14/170 (8.2)	3/59 (5.1)	11/111 (9.9)	0.384
Vaginal trauma	9/192 (4.7)	3/65 (4.6)	6/127 (4.7)	1.000
Premature placental abruption	1 (0.4)	1 (1.2)	0 (0.0)	0.300
Induced labor	83 (30.0)	23 (27.7)	60 (30.9)	0.695
Chorioamnionitis	5 (1.8)	2 (2.4)	3 (1.5)	0.638
Failure to progress/cephalopelvic disproportion	27/227 (11.9)	7/74 (9.5)	20/153 (13.1)	0.569
Instrumental delivery	15/192 (7.8)	3/65 (4.6)	12/127 (9.4)	0.370

* *Postpartum Hemorrhage; †Considered as elevated blood pressure levels during pregnancy: recorded cases of pre-eclampsia, gestational hypertension, or chronic hypertension; ‡Excluding those who did not go into labor or those without records.*

Out of the 277 participants in the sample, 29 had no record of risk stratification for PPH. Among the remaining participants, 248 had their risk stratification for PPH at the time of admission. Of these, 54.4% (n=135) were classified as green, indicating low risk for developing PPH, 44.4% (n=110) as yellow, indicating moderate risk, and 1.2% (n=3) as red, indicating high risk for developing PPH. Among the postpartum women who had PPH, 48.2% were classified as green, 38.6% as yellow, and 13.3% were not stratified. Risk stratification was not statistically associated with the occurrence of PPH.

Regarding the occurrence of bleeding, it was increased in 23.1% (n=64) of the participants, with two (0.7%) requiring blood transfusion. Decreased uterine tone was observed in 11.9% (n=33) of cases. In terms of management approaches used for PPH, oxytocin administration was the most prevalent, occurring in 98% of cases (n=82). Misoprostol was used in 55.4% (n=46) of cases, methylergometrine in 28.9% (n=24), tranexamic acid in 18.1% (n=15), uterine massage in 27.7% (n=23), and compressive uterine suturing using the B-Lynch technique in 2.4% (n=2).

Table 4 presents the variables related to birth. There was a significant association between the occurrence of PPH and the classification of newborn weight. Mothers with newborns classified as large for gestational age had a higher frequency of PPH.

**Table 4:** Bivariate analysis of variables related to birth and characteristics of newborns in the total sample and according to the presence of Postpartum Hemorrhage, Porto Alegre, Rio Grande do Sul, Brazil, 2020

Variables	Total sample (N=277; 100%)	With PPH^*^ (n=84; 29.8%)	Without PPH^*^ (n=198; 70.2%)	*p* value
	n (%)	n (%)	n (%)	
Umbilical cord clamping				0.771
Early	106 (37.9)	33 (39.8)	73 (37.1)	
Timely	174 (62.1)	50 (60.2)	124 (62.9)	
Breastfeeding within the first hour of life	146 (52.0)	43 (51.2)	103 (52.3)	0.970
Classification of newborn weight				0.023
Small for gestational age (SGA)^€^	38 (13.5)	8 (9.5)	30 (15.2)	
Appropriate for gestational age (AGA)^±^	224 (79.4)	65 (77.4)	159 (80.3)	
Large for gestational age (LGA)^£^	20 (7.1)	11 (13.1) ^†^	9 (4.5)	
Classification of gestational age				0.117
Preterm	27 (9.6)	4 (4.8)	23 (11.6)	
Term	255 (90.4)	80 (95.2)	175 (88.4)	

* *Postpartum Hemorrhage; €Small for Gestational Age; ±Appropriate for Gestational Age; £Large for Gestational Age; †Statistically significant association determined by the adjusted residual test at a significance level of 5%.*

The variables with a p-value < 0.20 in the bivariate analysis were included in the Multivariate Poisson Regression model (Table 5).

**Table 5:** Multivariate Poisson Regression Analysis to assess independently associated factors with Postpartum Hemorrhage, Porto Alegre, Rio Grande do Sul, Brazil, 2020

Variables	PR^€^	95% CI^*^	*p* value
Uterine distention	2.34	1.63 - 3.36	**<0.001**
Shock index ≥ 0.9	1.61	1.07 - 2.43	0.023
Elevated blood pressure levels during pregnancy	0.48	0.20 - 1.11	0.087
Gestational age classification (preterm)	0.70	0.29 - 1.69	0.425
Onset of labor	1.17	0.52 - 2.67	0.705
Vaginal delivery	1.97	0.88 - 4.41	0.098
Previous cesarean section	0.73	0.35 - 1.51	0.397
Education level			
Incomplete elementary education	3.51	0.46 - 26.8	0.226
Completed elementary education	5.22	0.70 - 39.0	0.107
Incomplete high school education	4.28	0.57 - 32.2	0.158
Completed high school education	4.79	0.66 - 35.0	0.123
Incomplete college education	4.71	0.60 - 37.3	0.142
Completed college education	1.00		

€PR - Prevalence Ratio;

* CI - Confidence Interval.

It is worth mentioning that the variables “use of forceps” (p=0.146) and “retained placenta” (p=0.113) were not included in the multivariate analysis as they were only considered for postpartum women who had a vaginal delivery. The variables “use of intravenous oxytocin (IV)” (p<0.001), “use of intramuscular oxytocin (IM)” (p=0.090), “prescription of intramuscular oxytocin (IM)” (p=0.170), and “curettage” (p=0.046) were also not included in the multivariate analysis, as these variables represent potential management approaches for PPH. The variable “classification of newborn weight” (p=0.023) was also not included in the multivariate analysis since all participants with newborns classified as LGA had a positive uterine distention variable, which was included in the multivariate analysis.

After adjusting for other variables, only the shock index and uterine distention remained statistically associated with PPH. Postpartum women with a shock index ≥ 0.9 had a 61% higher prevalence of PPH (relative risk [PR] = 1.61; 95% confidence interval [CI]: 1.07 - 2.43). Furthermore, postpartum women with uterine distention had a 134% higher prevalence of PPH (PR=2.34; 95% CI: 1.63 - 3.36), indicating them as intrapartum and antepartum risk factors, respectively, for the occurrence of PPH.

## DISCUSSION

A significant proportion of the postpartum women included in this study were diagnosed with PPH. Among the variables analyzed, uterine distention and shock index > 0.9 were found to be associated with the development of primary PPH. As previously mentioned, PPH is considered one of the leading causes of maternal mortality in Brazil, second only to hypertensive disorders^(7)^. These findings emphasize the importance of closely monitoring PPH and, when risk factors are identified, the healthcare team can collaborate to prevent complications related to this obstetric emergency.

Identifying risk factors for PPH is an ongoing crucial measure in obstetric care ^(4),^ as the clinical condition of postpartum women can change ^(4)^. The risk stratification used in this study was conducted at the time of hospital admission and is not directly linked to the diagnosis of PPH. It is important to note that the progression of labor and adverse situations during delivery can trigger PPH. Early identification of new risk factors will enable revised care planning, facilitating the early implementation of preventive measures for PPH.

In the northeastern region of Brazil, PPH is the second leading cause of admission to obstetric intensive care units, with 27.1% of hospitalized patients experiencing hemorrhagic shock^(8)^. Analyzing cases of PPH in vaginal deliveries in another region of Brazil, a study conducted at the Women’s Hospital in Campinas, São Paulo (SP), found that 31% of postpartum women had bleeding exceeding 500 ml, while 8.2% had bleeding exceeding 1,000 ml. Blood loss was measured by summing the volume collected in the surgical field using a blood collector, along with the weight of gauze, compresses, and pads used within 24 hours after delivery^(9)^. These findings align with the results of the present study, where 30% of the sample had primary PPH according to medical records.

The findings of this study, along with national data reflecting the PPH situation, when compared to international data, emphasize the need for improvements in Brazil. Examining the prevalence of PPH in three hospitals located in the Southern region of Ethiopia, a rate of 16.6% of primary PPH was identified^(10)^. A case-control study conducted at a hospital in Peru, involving 932 cases and 2,779 controls out of a total of 42,594 deliveries attended from 2000 to 2015, found a prevalence of 2.19% of PPH among postpartum women^(11)^.

Low prevalence rates were also identified in Guayaquil, Ecuador, between 2017 and 2018, where 7% of the 2,352 recorded births in 2017 were affected by PPH^(12)^. Similarly, in Switzerland, PPH occurred in 3.1% of postpartum women who gave birth between January 1993 and December 2014. This analysis was based on a database encompassing approximately 40% of all births in Switzerland^(13)^.

An analysis of a United States database tracking national estimates of hospital admissions revealed the occurrence of PPH in cesarean deliveries from 2011 to 2012. Non-severe PPH was observed in 15.7 per 1,000 admissions, PPH requiring blood transfusion in 5.0 per 1,000 admissions, and PPH necessitating interventions for control in 3.4 per 1,000 admissions^(14)^.

The prevalence of primary PPH in international settings is lower compared to Brazil and the findings of this study. This variability in PPH occurrence rates across different countries or even within different regions of the same country may be attributed to exposed inequities or barriers that restrict access to healthcare services. Factors such as limited healthcare access, low socioeconomic status, and delivery attended by unqualified professionals^(15)^ may closely correlate with adverse outcomes, as seen with PPH.

The occurrence of PPH can lead to various outcomes. However, early diagnosis and management are crucial in order to prevent complications and maternal mortality. In a multicenter study conducted across 27 healthcare centers in Brazil, out of 9,555 cases of severe maternal morbidity, 12.5% of postpartum women experienced complications related to PPH. Overall, PPH accounted for 23.5% of cases of maternal near miss and 21.4% of maternal deaths. Severe maternal outcomes (defined as the sum of maternal death and maternal near miss) were reported as 2.6 per 1,000 live births in postpartum women with PPH. Maternal age, gestational age, cesarean section, and previous uterine scar were identified as primary risk factors for severe maternal outcomes secondary to PPH^(16)^. These findings align with the present study, as no association was found between factors such as cesarean section or previous cesarean section and the occurrence of PPH.

Regarding the causes of maternal near miss, a study conducted in China from 2012 to 2017 across 18 hospitals in the Zhejiang province identified PPH as the primary cause, present in 76.3% of the records. The authors of the study argue that cases of maternal near miss were highly significantly associated with multiple pregnancies^(17)^, which supports the findings of this study, indicating a 134% higher prevalence of PPH among postpartum women with uterine distension.

In the United States, a study analyzing a national database of hospital admissions revealed a 60% increase in cases of PPH due to uterine atony when comparing two time periods (2001-2002 to 2011-2012). Additionally, there was an increase in the prevalence of PPH requiring blood transfusion in cesarean deliveries, rising from 2.1 to 5.0 per 1,000 complicated hospitalizations^(14)^.

The results of the study emphasize the importance for physicians and healthcare institutions to better understand this obstetric emergency. Identifying risk factors and involving a multidisciplinary team can help reverse the increasing number of PPH cases and prevent this complication^(14)^. Research studies that provide data over a period of time, like this one, are valuable as they capture temporal trends.

Regarding the most severe outcome of PPH - maternal death - maternal mortality rates in Brazil varied across regions from 1996 to 2016: Northeast, 34.5%; Southeast, 31.0%; South, 16.5%; North, 10.8%; and Midwest, 7.2%^(18)^. In Juiz de Fora, Minas Gerais, the epidemiological profile of maternal mortality, based on the analysis of Confidential Maternal Death Investigation Forms, revealed that 14.1% of all maternal deaths between 2005 and 2015 were due to hypovolemic shock^(19)^. Some authors argue that most factors contributing to maternal mortality are related to PPH, hypertensive disorders, and diseases developed during the pregnancy-postpartum period, and appropriate healthcare could prevent the majority of these factors^(20)^.

Considering that maternal death often stems from causes that could be prevented during the pregnancy-postpartum period, there is a significant disparity in maternal mortality rates between developed countries (1 in 100,000 births) and developing countries (1 in 1,000 births), underscoring the influence of healthcare infrastructure and attention to health^(21)^.

Several countries exemplify this disparity, such as Turkey, where the maternal mortality ratio (MMR) was 19.7 per 100,000 live births from 2012 to 2015^(22)^. In Brazil, a study compares MMR indicators and their trends over the years, revealing that in 1990, the MMR was 143.2 per 1,000 live births, declining to 59.7 per 1,000 live births by 2015^(23)^.

Regarding risk factors, uterine distension has shown statistically significant results and is considered a major risk factor for the occurrence of PPH. Evidence has demonstrated that uterine distension predisposes to uterine atony^(24)^, which is considered the leading cause of PPH^(4)^. Uterine distension during pregnancy can occur in cases of multiple fetuses, large-for-gestational-age infants (LGA), or when there is an increase in amniotic fluid (polyhydramnios)^(4)^.

Supporting the findings of this study, research conducted in Spain illustrates the association between infants weighing over 4000g and the occurrence of PPH^(25)^. A retrospective cohort study conducted in Peru from 2015 to 2017 showed that multiple gestation is a risk factor for PPH: 19% of multiple gestations had PPH due to uterine atony, compared to 7% in singleton pregnancies, concluding that the risk of developing PPH due to uterine atony is 2.75 times higher in postpartum women with multiple gestations compared to those with singleton pregnancies^(26)^.

Multiple pregnancy was also identified as a risk factor for severe PPH in Norway, where 1,064 cases of severe PPH (defined as blood loss ≥1500 ml or the need for postpartum blood transfusion) were identified from 2008 to 2011. The most common etiology for severe PPH was uterine atony, reported in 60.4% of cases, followed by placental complications in 36%^(27)^.

Regardless of the mode of delivery, uterine atony was considered the leading cause of PPH in a study conducted at a hospital in Peru, where an association between fetal macrosomia and PPH was also observed^(11)^. In Suriname, authors analyzed hospital deliveries in 2017 and identified multiple gestation and fetal macrosomia as risk factors for severe PPH (blood loss ≥1,000 ml or ≥500 ml associated with hypotension or transfusion of at least three units of blood products)^(28)^.

Multiple gestation and fetal macrosomia were mentioned as the second and third most prevalent risk factors for PPH among all vaginal deliveries occurring between October and December 2003 and October and December 2005 in 19 public maternity hospitals in Argentina and Uruguay. The study observed a rate of 10.8% for moderate PPH (blood loss of at least 500 ml) and 1.9% for severe PPH (blood loss of at least 1,000 ml)^(29)^.

In this study, another factor statistically associated with PPH was the shock index (SI), a clinical parameter that can be used to assess the impact of obstetric blood loss and assist in the choice of management strategies for PPH. The SI is calculated by dividing the heart rate by the systolic blood pressure, reflecting the hemodynamic state. It is considered an easily performed method and useful in predicting the need for massive transfusion. SI values ≥ 0.9 indicate significant blood loss and the potential need for blood transfusion during PPH management^(4,30)^.

SI values ≥ 0.9 can be utilized to identify postpartum women who require urgent high-level care. The SI proves to be more effective in evaluating the hemodynamic state and cardiovascular compromise compared to assessing vital signs in isolation (heart rate and systolic blood pressure)^(31)^. Importantly, an SI > 0.9 can serve as a predictor for the need for blood transfusion and can predict adverse outcomes of PPH^(32)^. Visual estimation of blood loss and isolated vital signs are not sufficient for recognizing PPH. Therefore, methods for identifying PPH should be easily applicable and include the patient’s clinical findings, aiding in early diagnosis and timely treatment. The SI is a useful tool for early identification of postpartum women at risk of cardiovascular alterations and provides better prediction compared to other isolated vital signs. It is considered an easily calculable parameter, utilizing the patient’s clinical signs, and serves as an early indicator of severity, although additional evaluation is necessary in cases of PPH^(33)^.

A study based on the review of clinical records of 105 postpartum women diagnosed with obstetric hemorrhage in an Intensive Care Unit found that an obstetric SI ≥ 0.9 was associated with an average blood loss of 3,000 ml. Among the patients included in the study, 65 (61%) had an SI result above 0.9, and 38 (58%) required massive transfusion. An SI value ≥ 0.9 predisposed to severe complications such as acute renal failure and infectious processes^(34)^. In this research, 2.4% of postpartum women diagnosed with PPH required blood transfusion, showing a discrepancy compared to the previous study.

The occurrence of blood transfusion is essential in managing PPH, although determining the appropriate type and quantity of blood components needed can be challenging. This difficulty arises from the physiological changes in blood pressure levels, vascular tone, and circulating volume experienced by pregnant women, which can impede early diagnosis and recognition of hemorrhage symptoms. It is in this context that calculating the shock index (SI) becomes crucial^(34)^.

A cohort study conducted in Campinas assessed blood loss by weighing the surgical field, dressings, and sanitary pads used within the first 24 hours after delivery. The data revealed that 44.5% of postpartum women experienced bleeding exceeding 500 ml during this period. However, this bleeding did not have clinical implications for the patients. The study indicated that the SI parameter exhibited high specificity but low sensitivity, suggesting that vital signs alone, such as heart rate and systolic blood pressure, have limited ability to promptly identify increased bleeding. Nevertheless, given the high specificity of the SI, values below the defined cutoff points can be used to rule out increased vaginal bleeding, while higher values can serve as an alert for heightened postpartum bleeding. This approach aims to identify postpartum women who require additional attention or referral for further treatment, particularly in lower-level healthcare settings^(35)^.

Inconsistencies in diagnosing PPH can make it challenging to estimate its incidence. Clinical estimation methods, such as weighing dressings and visual assessment, are imprecise, often underestimating blood loss in vaginal deliveries and overestimating it in cesarean sections^(36)^.

The American College of Obstetricians and Gynecologists recommends the use of quantitative methods to measure blood loss during delivery, as they provide more accurate data compared to visual estimation^(37)^. However, in the present study, the approach of the management team to control PPH, based on the care protocol, was considered, as the medical records consulted did not contain a definitive diagnosis of PPH, leading to imprecision in the records.

Considering the likelihood of PPH occurrence, the support actions during delivery, and the potential interventions required, it is suggested that healthcare professionals and services possess knowledge and base their practices on evidence to ensure less harmful outcomes for postpartum women. Educational initiatives focused on technology are increasingly necessary to enhance clinical practice^(38)^.

### Study limitations

The study has limitations associated with the lack of standardized documentation of PPH cases in the medical records of postpartum women at the institution, which posed challenges in classifying and categorizing the results. These limitations were mitigated by analyzing and discussing the various recording methods with experts in the field, leading to consensus on the categorization of the medical record findings. Furthermore, it is important to note that data collection and categorization were performed by the study’s principal researcher, minimizing confounding biases and interpretation biases.

### Contributions to the nursing field

This study provides valuable insights into the prevalence of primary PPH in a university hospital, identifies risk factors, and highlights the diverse recording practices for this complication within the institution. Findings like these can prompt healthcare professionals to recognize the significance of accurate record-keeping in their daily practice and work towards standardizing recording protocols. The development of an objective and standardized record, encompassing clinical information and a precise diagnosis of PPH based on accurate measurement of postpartum blood loss, could enhance healthcare delivery for this population and inform continuous care efforts.

The study found a high prevalence of primary PPH compared to evidence from previous studies. This outcome stimulates important discussions in clinical practice about strategies for early identification of risk factors, optimal management approaches, and the implementation of preventive care measures, ultimately aiming to improve the quality of care and reduce maternal morbidity and mortality rates in accordance with international recommendations.

Although not statistically significant, it is crucial not to disregard certain risk factors in clinical practice, as they have been identified in other studies. Additionally, the limited evidence on risk factors related to the studied outcome, coupled with the absence of a quantitative tool for optimizing blood loss measurement, underscores the ongoing need for further investigation in this field.

## CONCLUSIONS

The findings revealed a high prevalence of PPH compared to results from other studies. However, it is important to note that the prevalence rate of PPH in the study population was determined in conjunction with records of increased bleeding and/or records of care interventions for PPH control, based on the institution’s care protocol.

Statistically significant associations were found between uterine distension and the Shock Index (SI). Although these factors have been previously associated with PPH occurrence in different research and clinical contexts, reaffirming their significance is necessary to enhance clinical practice and employ predictive resources for PPH occurrence.

It is worth noting that, although risk stratification is a means of assessing postpartum women at a higher risk of developing PPH, this variable was not statistically associated with the occurrence of the outcome in the study sample.

It is suggested to develop a standardized record or indicator for PPH cases in collaboration with managers and the multidisciplinary team, aiming to standardize the identification and subsequent recording of PPH cases based on scientific evidence. Standardized records can provide information that allows for research such as this, serving as examples for improving hospital records. Continued education is a way to empower the care team regarding the identification of PPH and the importance of accurate record-keeping, not only in these cases but in all aspects of care provided during hospitalization.

It is necessary to raise awareness among professionals about the importance of complete, homogeneous, qualified, and standardized records to avoid compromising the quality of care and the conduct of studies that use electronic medical records as a database.

## Supplementary Material

0034-7167-reben-76-05-e20220134-sup01

## Data Availability

https://doi.org/10.48331/scielodata.PSGIMB
