# Supplementary material for: Prevalence of risk factors for primary postpartum hemorrhage in a university hospital
Source: Rev Bras Enferm. 2023 Nov 27;76(5):e20220134. doi: 10.1590/0034-7167-2022-0134 (PMC10680393; doi:10.1590/0034-7167-2022-0134)
Supplement: 0034-7167-reben-76-05-e20220134-sup01 [file 0034-7167-reben-76-05-e20220134-sup01.pdf]

NUMERO	IDADE	IDADECAT	RACA	ESCOL	PN	CONSULTA	CONSULTA	IG
P188	35	3	3	2	1	7	1	39+5
P218	27	2	2	4	1	7	1	40
P258	43	3	1	7	1	10	2	36+6
P37	29	2	1	3	1	2	1	41
P187	21	2	1	5	1	5	1	38+6
P03	25	2	1	4	1	4	1	40
P05	33	2	1	3	1	7	1	39+4
P06	29	2	1	5	1	18	2	39+3
P07	25	2	1	5	1	10	2	39
P08	32	2	1	5	1	7	1	38+4
P09	24	2	1	5	1	12	2	40+6
P10	25	2	1	5	1	10	2	39
P100	31	2	1	5	1	12	2	39+5
P101	37	3	1	2	1	10	2	38
P103	32	2	2	6	1	4	1	40
P104	24	2	1	6	1	8	2	34+3
P105	27	2	1	4	1	2	1	40+5
P106	26	2	1	5	1	11	2	38+4
P107	19	1	1	4	1	2	1	36+5
P109	22	2	1	6	1	11	2	40+5
P11	33	2	1	5	1	10	2	39+4
P111	34	2	1	5	1	10	2	39
P115	35	3	1	7	1	9	2	36+4
P116	28	2	1	2	1	10	2	40+5
P117	23	2	1	6	1	12	2	38+4
P118	31	2	1	3	1	6	1	40+5
P119	16	1	1	2	1	4	1	37+6
P12	25	2	1	2	1	5	1	38+5
P121	21	2	3	4	1	9	2	38+2
P122	18	1	1	5	2	88	88	38+5
P124	24	2	1	5	1	11	2	40+2
P125	32	2	1	5	1	10	2	39
P127	28	2	1	2	1	5	1	38+4
P128	21	2	1	2	1	5	1	40+6
P129	32	2	1	5	1	8	2	40+6
P13	29	2	2	4	1	11	2	40
P130	26	2	1	4	1	10	2	36+5
P132	36	3	1	2	1	8	2	39
P135	25	2	1	5	1	6	1	39+4
P136	17	1	3	2	1	5	1	39
P138	18	1	2	4	2	88	88	39
P139	29	2	2	5	1	11	2	41
P14	25	2	1	5	1	9	2	39+5
P140	30	2	1	5	1	11	2	39+4
P141	25	2	1	5	1	8	2	39+3
P143	25	2	1	5	1	9	2	40+5
P144	27	2	1	5	1	9	2	41+1
P146	23	2	1	5	1	6	1	40
P148	29	2	2	5	1	9	2	39+6

P149	20	2	1	2	1	9	2	37+5
P15	18	1	1	3	1	10	2	39
P150	20	2	1	3	1	5	1	40+2
P151	16	1	2	2	1	3	1	38+3
P152	21	2	1	2	1	11	2	37+6
P154	23	2	2	4	1	4	1	36+1
P156	18	1	1	3	1	7	1	36+6
P157	18	1	1	4	1	13	2	39+6
P161	26	2	2	6	1	10	2	40
P162	37	3	2	3	1	7	1	38+5
P163	23	2	1	4	1	13	2	38+4
P164	27	2	1	3	1	15	2	40+4
P165	21	2	2	4	1	8	2	38
P166	21	2	1	5	1	9	2	40+1
P167	18	1	1	3	1	4	1	40+5
P168	21	2	1	2	1	1	1	40
P169	18	1	1	4	1	5	1	38
P17	23	2	3	5	1	8	2	40
P170	23	2	2	5	1	9	2	38
P172	20	2	1	3	1	8	2	38+6
P173	32	2	1	5	2	88	88	41+1
P174	19	1	3	5	1	9	2	40+3
P176	18	1	1	3	1	6	1	39
P177	26	2	2	6	1	10	2	40
P178	19	1	3	2	1	6	1	36+2
P18	39	3	2	5	1	20	2	36+3
P181	34	2	1	4	1	10	2	37+1
P182	28	2	3	2	2	88	88	40+1
P184	38	3	1	7	1	6	1	37+2
P185	24	2	1	6	1	10	2	38+2
P189	28	2	1	7	1	19	2	39+6
P191	31	2	1	5	1	8	2	39+3
P192	23	2	1	3	1	11	2	38+6
P195	25	2	1	5	1	8	2	41+3
P196	18	1	1	2	1	7	1	41
P197	32	2	2	3	1	11	2	40+6
P198	23	2	1	4	1	8	2	39+1
P20	23	2	2	2	2	88	88	35+6
P200	23	2	2	5	1	8	2	38+6
P201	38	3	1	5	1	12	2	38+2
P202	19	1	1	3	1	8	2	40+3
P203	22	2	3	2	2	88	88	40
P204	19	1	2	5	1	9	2	39+5
P205	21	2	1	5	1	7	1	39+3
P206	29	2	1	2	1	3	1	39+4
P207	26	2	1	4	1	10	2	38
P21	29	2	1	5	1	10	2	40+6
P210	22	2	1	5	1	14	2	39+6
P211	28	2	1	7	1	9	2	40
P212	37	3	1	5	1	15	2	40+3

P213	23	2	2	4	1	5	1	39+3
P214	27	2	1	5	1	8	2	37+6
P215	36	3	1	7	1	9	2	39+2
P217	32	2	1	6	1	12	2	38+4
P22	36	3	1	5	1	6	1	40+1
P221	24	2	2	5	1	6	1	40
P222	30	2	1	3	1	3	1	35+1
P223	25	2	1	5	1	7	1	37
P224	32	2	1	5	1	10	2	38+2
P225	39	3	1	5	1	14	2	39+2
P226	24	2	1	2	1	7	1	40+1
P227	30	2	1	5	1	8	2	39+5
P228	21	2	1	2	1	6	1	37
P229	20	2	3	4	2	88	88	40+3
P23	28	2	1	5	1	4	1	40
P230	23	2	3	3	1	12	2	41
P231	33	2	1	3	1	13	2	39+3
P232	26	2	1	3	1	10	2	39+6
P233	21	2	1	2	1	14	2	40+3
P234	36	3	1	5	1	5	1	38+1
P235	31	2	1	5	2	88	88	38+3
P238	38	3	1	5	1	9	2	38
P239	21	2	1	6	1	7	1	41
P24	15	1	2	2	1	4	1	39+4
P240	19	1	1	2	1	13	2	40+5
P241	21	2	1	5	1	14	2	38+4
P246	31	2	1	4	1	7	1	39
P247	37	3	1	5	1	4	1	39+3
P248	29	2	1	3	1	12	2	40+5
P249	18	1	1	4	1	12	2	40
P25	19	1	1	5	1	8	2	39+4
P250	26	2	1	4	1	4	1	39+1
P251	27	2	1	3	1	5	1	38
P252	36	3	1	2	1	4	1	40+3
P253	30	2	1	5	1	16	2	39
P254	40	3	1	7	1	8	2	36+6
P255	33	2	1	4	1	8	2	39
P26	22	2	1	5	1	9	2	37+5
P261	39	3	1	5	1	5	1	39+2
P262	32	2	2	2	1	3	1	39
P263	26	2	3	4	1	13	2	39+2
P264	36	3	1	5	1	7	1	39
P267	16	1	2	2	1	11	2	40+1
P268	29	2	1	4	1	12	2	38+6
P27	22	2	2	2	1	9	2	38+4
P270	33	2	1	3	1	8	2	39+5
P272	33	2	1	5	1	7	1	39
P273	23	2	1	4	1	8	2	39+6
P274	28	2	2	2	1	11	2	39+5
P275	35	3	1	2	1	11	2	39

P28	23	2	1	5	1	11	2	40+6
P31	24	2	1	3	1	6	1	40+3
P32	31	2	1	2	1	1	1	39+5
P33	20	2	1	6	1	7	1	41+1
P35	27	2	1	5	1	10	2	38+2
P36	29	2	1	6	1	7	1	37+6
P39	25	2	3	4	1	13	2	39+1
P41	27	2	3	2	1	11	2	40+1
P42	25	2	3	2	1	11	2	36+2
P44	27	2	1	5	1	10	2	38+5
P45	25	2	1	4	1	3	1	39+5
P46	36	3	1	5	1	11	2	39
P47	25	2	2	2	1	8	2	39+2
P48	33	2	1	5	1	14	2	39+3
P49	27	2	3	3	1	1	1	38+6
P51	24	2	3	4	1	18	2	38+4
P55	20	2	1	5	2	66	66	38+2
P57	27	2	1	5	1	11	2	38+6
P59	35	3	1	6	1	10	2	41
P60	24	2	1	2	1	10	2	36+5
P62	22	2	1	5	1	8	2	39+2
P63	19	1	1	4	1	6	1	39+2
P64	42	3	1	5	1	12	2	40+3
P67	27	2	1	2	2	66	66	39+5
P68	17	1	2	2	1	6	1	38+1
P69	19	1	1	3	1	10	2	38+2
P70	22	2	1	3	1	8	2	38+3
P72	25	2	1	6	1	8	2	37+4
P73	35	3	1	6	1	7	1	38+6
P74	38	3	1	7	1	10	2	38+1
P76	24	2	2	2	1	5	1	38+5
P77	25	2	1	5	1	11	2	40
P79	28	2	2	5	1	5	1	36+6
P80	32	2	2	5	1	6	1	34+5
P83	26	2	1	4	1	6	1	40+5
P84	26	2	1	4	1	13	2	41
P86	28	2	1	5	1	8	2	40+6
P92	23	2	1	5	1	10	2	40+3
P93	26	2	1	5	1	5	1	39+2
P95	22	2	1	5	1	10	2	39
P97	20	2	1	4	1	9	2	41+1
P98	18	1	1	3	1	11	2	40
P99	31	2	1	7	1	9	2	38+3
P01	21	2	1	3	1	19	2	39+1
P02	32	2	2	3	1	12	2	36+5
P04	28	2	1	5	1	4	1	39+2
P102	30	2	2	4	1	9	2	40+2
P108	29	2	3	5	1	11	2	38+1
P110	28	2	1	5	1	11	2	40+4
P112	29	2	2	3	1	7	1	39

P113	26	2	2	2	1	3	1	41+2
P114	30	2	1	5	1	7	1	39+3
P120	19	2	1	5	1	9	2	39+6
P123	18	1	1	2	1	9	2	40
P126	34	2	1	5	1	8	2	37+2
P131	29	2	1	5	1	13	2	40+1
P133	36	3	2	4	1	8	2	37
P134	31	2	1	5	1	13	2	39
P137	24	2	1	5	1	12	2	41
P142	32	2	2	5	1	10	2	41
P145	26	2	1	5	1	11	2	38+2
P147	29	2	2	3	1	11	2	39
P153	23	2	1	5	1	23	2	40+1
P155	25	2	2	5	1	12	2	39+1
P158	23	2	1	5	1	12	2	40+1
P159	22	2	1	4	1	5	1	36
P16	28	2	2	4	1	9	2	36+2
P160	23	2	1	3	1	10	2	39+5
P171	21	2	1	2	1	11	2	40+1
P175	20	2	1	5	1	9	2	39
P179	28	2	1	5	1	11	2	39
P180	18	1	1	3	1	9	2	39+4
P183	39	3	1	2	1	12	2	37+4
P186	26	2	2	6	1	18	2	41
P190	19	1	2	4	1	4	1	41+4
P193	29	2	1	5	1	7	1	40+2
P194	24	2	3	3	1	14	2	41+1
P199	36	3	1	5	1	18	2	37+1
P19A	31	2	1	5	1	16	2	37
P19B	88	88	88	88	88	88	88	88
P208	42	3	1	5	1	15	2	37+6
P209	29	2	1	5	1	13	2	38+5
P216	15	1	3	2	1	8	2	40
P219	21	2	1	2	1	6	1	40+1
P220	34	2	1	4	1	16	2	40+5
P236	32	2	2	5	1	12	2	41
P237	23	2	1	6	1	12	2	39+5
P242	27	2	1	2	1	10	2	39+3
P243	34	2	1	5	1	9	2	39+3
P244	17	1	1	4	1	7	1	41+1
P245	27	2	1	2	1	4	1	37+6
P256	28	2	2	2	1	14	2	39+3
P257	17	1	1	3	1	13	2	40+3
P259	25	2	2	2	1	11	2	40
P260	24	2	1	5	1	12	2	40+5
P265	27	2	1	5	1	14	2	40+5
P266A	28	2	2	3	1	10	2	37
P266B	88	88	88	88	88	88	88	88
P269A	30	2	1	7	1	5	2	35+1
P269B	88	88	88	88	88	88	88	88

P271	25	2	1	3	1	10	2	39
P276	26	2	1	5	1	17	2	37+3
P277	23	2	1	2	2	88	88	40+4
P29	42	3	1	5	1	12	2	39
P30	16	1	1	2	1	11	2	39+4
P34A	22	2	3	4	1	10	2	36
P34B	88	88	88	88	88	88	88	88
P38	23	2	1	5	1	7	1	38
P40	37	3	1	7	1	9	2	40+1
P43A	29	2	1	4	2	66	66	35+3
P43B	88	88	88	88	88	88	88	88
P50	37	3	1	5	1	22	2	40+4
P52	24	2	1	4	1	10	2	36+1
P53	33	2	1	7	1	7	1	37+4
P54	23	2	1	5	1	12	2	37+2
P56	25	2	1	4	1	11	2	39+5
P58	18	1	2	2	1	9	2	40+4
P61	38	3	1	7	1	12	2	39+6
P65	35	3	1	5	1	15	2	39
P66	25	2	3	2	1	8	2	38
P71	37	3	1	5	1	10	2	36
P75	38	3	1	5	1	6	1	38+1
P78	31	2	1	4	1	11	2	40+6
P81	29	2	2	3	1	4	1	39
P82	26	2	1	5	1	11	2	40+6
P85	37	3	1	7	2	66	66	38
P87	30	2	2	2	1	11	2	41+1
P88	40	3	1	7	1	6	1	39
P89	40	3	1	7	1	9	2	39
P90	29	2	2	5	1	10	2	40+3
P91	38	3	1	5	1	8	2	38
P94	23	2	3	5	1	2	1	39+6
P96	36	3	1	3	1	6	1	36+3

COMPGA	COMPGAH	COMPGAD	COMPGATI	COMPGAH	COMPGASI	COMPGAH	COMPGACI	COMPGAPI
1	2	1	2	2	2	2	2	2
1	2	2	2	2	1	2	2	2
1	2	2	2	1	2	1	1	1
2	88	88	88	88	88	88	88	88
1	2	2	2	2	2	2	2	2
2	88	88	88	88	88	88	88	88
1	2	2	2	2	1	2	2	2
1	2	2	2	2	2	2	2	2
1	2	1	2	2	2	2	2	2
1	2	1	2	2	2	2	2	2
2	88	88	88	88	88	88	88	88
1	2	1	2	2	2	2	2	2
1	2	2	2	1	2	2	2	2
1	2	2	2	2	2	2	2	2
2	88	88	88	88	88	88	88	88
1	2	1	2	2	2	2	2	2
1	2	2	2	2	1	2	2	2
1	2	1	2	2	2	2	2	2
1	2	2	2	2	2	2	2	2
2	88	88	88	88	88	88	88	88
2	88	88	88	88	88	88	88	88
1	2	2	2	1	2	2	2	2
1	2	2	2	2	2	2	2	2
2	88	88	88	88	88	88	88	88
1	2	2	2	2	2	2	1	2
2	88	88	88	88	88	88	88	88
2	88	88	88	88	88	88	88	88
1	2	2	2	2	2	2	2	2
1	2	2	2	1	2	2	2	2
1	2	2	2	2	2	2	2	2
1	2	2	2	2	2	2	2	2
1	2	2	2	2	2	2	2	1
1	2	2	2	2	2	2	2	2
2	88	88	88	88	88	88	88	88
1	2	2	2	2	2	2	2	2
2	88	88	88	88	88	88	88	88
1	1	2	2	2	2	2	2	1
1	2	2	2	2	2	1	2	2
2	88	88	88	88	88	88	88	88
1	2	2	2	2	2	2	2	2
2	88	88	88	88	88	88	88	88
2	88	88	88	88	88	88	88	88
2	88	88	88	88	88	88	88	88
1	2	2	2	2	2	2	2	2
1	2	2	2	2	1	2	2	2
1	1	2	2	2	2	2	2	2
2	88	88	88	88	88	88	88	88
1	2	1	2	2	2	2	2	2
2	88	88	88	88	88	88	88	88

1	2	2	2	2	2	2	1	2
1	2	2	2	1	2	2	2	1
1	2	2	2	2	2	2	2	2
2	88	88	88	88	88	88	88	88
2	88	88	88	88	88	88	88	88
1	2	2	2	2	2	2	2	2
1	2	1	2	2	2	2	2	2
1	2	2	2	2	2	2	2	2
1	1	2	2	2	2	2	2	2
1	2	2	2	2	2	2	2	1
2	88	88	88	88	88	88	88	88
1	2	1	2	2	2	2	2	2
1	2	2	2	2	2	2	2	2
2	88	88	88	88	88	88	88	88
1	2	2	2	2	2	2	2	2
2	88	88	88	88	88	88	88	88
1	2	2	2	2	2	2	2	2
2	88	88	88	88	88	88	88	88
1	2	2	2	2	2	2	1	2
1	2	1	2	2	2	2	2	2
2	88	88	88	88	88	88	88	88
2	88	88	88	88	88	88	88	88
1	2	2	2	2	2	2	2	2
1	1	2	2	2	2	2	2	2
2	88	88	88	88	88	88	88	88
1	2	1	2	2	2	2	2	2
1	1	1	2	2	2	2	2	2
2	88	88	88	88	88	88	88	88
1	2	2	2	1	2	2	2	2
1	1	2	2	1	2	2	2	2
1	2	2	2	2	2	2	2	2
1	2	1	2	2	2	2	2	2
1	2	2	2	1	2	2	2	2
2	88	88	88	88	88	88	88	88
2	88	88	88	88	88	88	88	88
2	88	88	88	88	88	88	88	88
1	2	2	2	2	2	2	2	2
1	2	2	2	2	1	2	2	2
1	2	2	2	2	1	2	2	2
1	2	2	2	2	2	2	2	2
2	88	88	88	88	88	88	88	88
2	88	88	88	88	88	88	88	88
1	2	2	2	2	2	2	2	2
1	2	2	2	2	1	2	2	2
1	1	1	2	2	2	2	2	2
2	88	88	88	88	88	88	88	88
2	88	88	88	88	88	88	88	88
1	2	2	2	1	2	2	2	2
2	88	88	88	88	88	88	88	88
1	2	2	2	2	2	2	2	2

2	88	88	88	88	88	88	88	88
1	2	2	2	2	2	2	2	2
2	88	88	88	88	88	88	88	88
1	2	2	2	2	2	2	2	2
2	88	88	88	88	88	88	88	88
2	88	88	88	88	88	88	88	88
1	2	2	2	2	2	2	2	1
1	1	2	2	2	2	2	2	2
1	2	2	2	2	2	2	2	2
1	2	1	2	2	2	2	2	2
2	88	88	88	88	88	88	88	88
1	2	1	2	2	2	2	2	2
2	88	88	88	88	88	88	88	88
2	88	88	88	88	88	88	88	88
2	88	88	88	88	88	88	88	88
1	2	2	2	2	2	2	2	2
1	1	1	2	2	2	2	2	2
1	2	2	2	2	2	2	2	2
2	88	88	88	88	88	88	88	88
1	2	2	2	2	2	2	2	2
2	88	88	88	88	88	88	88	88
1	2	1	2	2	2	2	2	2
2	88	88	88	88	88	88	88	88
2	88	88	88	88	88	88	88	88
1	1	2	2	2	1	2	2	2
1	2	2	2	2	1	2	2	2
2	88	88	88	88	88	88	88	88
1	2	2	2	2	2	2	2	2
1	2	2	2	2	1	2	2	2
1	2	1	2	2	2	2	2	2
1	2	2	2	2	2	2	2	2
1	2	2	2	2	2	2	2	2
1	2	1	2	2	2	2	2	2
1	1	2	2	2	2	2	2	2
1	2	1	2	1	2	2	2	2
2	88	88	88	88	88	88	88	88
2	88	88	88	88	88	88	88	88
2	88	88	88	88	88	88	88	88
1	2	2	2	2	2	2	2	2
1	2	1	2	2	2	2	2	2
1	2	2	2	2	2	2	2	2
2	88	88	88	88	88	88	88	88
1	2	2	2	2	2	2	2	2
1	1	2	2	2	2	2	2	2
2	88	88	88	88	88	88	88	88
1	1	2	2	2	2	2	2	2
2	88	88	88	88	88	88	88	88
1	2	2	2	2	2	2	2	2
1	2	2	2	2	2	2	2	2
2	88	88	88	88	88	88	88	88

1	2	2	2	2	2	2	2	2
2	88	88	88	88	88	88	88	88
1	2	2	2	2	2	2	2	2
1	1	1	2	2	2	2	2	2
1	2	2	2	1	2	2	2	2
1	2	2	1	2	2	2	2	2
1	2	1	2	2	2	2	2	2
2	88	88	88	88	88	88	88	88
1	2	2	2	2	2	2	2	1
1	2	1	2	2	2	2	2	2
1	2	2	2	1	2	2	2	2
1	2	1	2	2	2	2	2	2
1	2	2	2	2	2	2	2	2
1	2	2	2	2	2	2	2	2
2	88	88	88	88	88	88	88	88
1	2	1	2	2	2	2	2	2
1	2	1	2	2	2	1	2	2
2	88	88	88	88	88	88	88	88
2	88	88	88	88	88	88	88	88
1	1	2	2	2	2	2	2	2
2	88	88	88	88	88	88	88	88
2	88	88	88	88	88	88	88	88
2	88	88	88	88	88	88	88	88
2	88	88	88	88	88	88	88	88
1	2	2	2	2	2	2	2	2
1	2	2	2	2	2	2	2	2
2	88	88	88	88	88	88	88	88
1	1	2	2	1	2	2	2	2
1	2	1	2	2	2	2	2	2
1	2	2	2	2	2	2	2	2
1	2	2	2	2	2	1	2	1
1	2	1	2	2	2	2	2	2
1	2	1	2	2	2	2	2	2
1	2	2	2	2	2	2	2	2
1	2	2	2	2	2	2	2	2
2	88	88	88	88	88	88	88	88
2	88	88	88	88	88	88	88	88
2	88	88	88	88	88	88	88	88
1	2	2	2	2	2	2	2	2
1	1	2	2	2	2	2	2	2
2	88	88	88	88	88	88	88	88
2	88	88	88	88	88	88	88	88
1	2	2	2	2	2	2	2	2
1	2	1	2	2	2	2	2	2
1	1	2	2	2	2	2	2	1
2	88	88	88	88	88	88	88	88
2	88	88	88	88	88	88	88	88
1	1	2	2	2	2	2	2	2
1	2	2	2	2	2	2	2	2
2	88	88	88	88	88	88	88	88

2	88	88	88	88	88	88	88	88
2	88	88	88	88	88	88	88	88
1	2	1	2	2	2	2	2	2
2	88	88	88	88	88	88	88	88
1	1	2	2	2	2	2	2	1
1	2	2	2	2	2	2	2	2
1	2	2	2	2	2	1	1	2
1	2	1	2	2	2	2	2	2
1	2	2	2	2	2	2	2	2
2	88	88	88	88	88	88	88	88
2	88	88	88	88	88	88	88	88
1	2	2	2	2	2	2	2	2
1	2	2	2	2	1	2	2	2
1	2	2	2	2	2	2	2	2
2	88	88	88	88	88	88	88	88
1	2	2	2	2	2	2	2	1
1	2	2	2	2	2	1	1	2
1	2	1	2	2	2	2	2	2
1	2	2	2	2	2	2	2	2
1	2	2	2	2	2	2	2	2
2	88	88	88	88	88	88	88	88
1	2	2	2	2	2	2	1	2
1	1	1	2	2	2	2	2	2
1	2	1	2	2	2	2	2	2
1	2	2	2	2	2	2	2	2
2	88	88	88	88	88	88	88	88
1	1	2	2	2	2	2	2	2
1	2	2	2	2	2	2	2	2
1	2	1	2	2	2	2	2	2
88	88	88	88	88	88	88	88	88
1	2	2	2	2	2	1	2	2
1	2	1	2	2	2	2	2	2
1	2	2	2	2	2	2	2	2
1	2	2	2	2	2	2	2	2
2	88	88	88	88	88	88	88	88
1	2	2	2	2	2	2	2	2
2	88	88	88	88	88	88	88	88
1	2	1	2	2	2	2	2	2
1	2	2	2	2	2	2	2	2
2	88	88	88	88	88	88	88	88
1	2	2	2	2	2	2	2	2
1	2	1	2	2	2	2	2	2
1	2	1	2	2	2	2	2	2
2	88	88	88	88	88	88	88	88
1	2	2	2	2	2	2	2	2
2	88	88	88	88	88	88	88	88
1	2	2	2	2	2	2	2	2
88	88	88	88	88	88	88	88	88
1	2	2	1	2	2	2	2	2
88	88	88	88	88	88	88	88	88

2	88	88	88	88	88	88	88	88
1	1	1	2	2	2	2	2	2
2	88	88	88	88	88	88	88	88
1	2	1	2	2	2	2	2	2
1	2	2	2	2	2	1	1	2
1	2	2	2	2	2	2	1	2
88	88	88	88	88	88	88	88	88
1	1	2	2	1	2	2	2	2
1	2	2	2	2	2	2	2	2
1	2	1	2	2	2	2	2	2
88	88	88	88	88	88	88	88	88
1	2	2	2	2	2	2	2	2
1	2	2	2	2	2	2	2	2
1	2	2	2	2	2	2	2	2
1	1	1	2	2	2	2	2	1
1	2	2	2	2	1	2	2	2
1	2	2	2	2	2	2	2	2
1	2	2	2	2	2	2	2	1
1	2	1	2	2	2	2	2	2
1	2	2	2	2	2	2	2	2
1	2	2	2	2	1	2	2	2
2	88	88	88	88	88	88	88	88
2	88	88	88	88	88	88	88	88
2	88	88	88	88	88	88	88	88
1	2	2	2	2	1	2	2	2
1	2	1	2	1	1	2	2	2
2	88	88	88	88	88	88	88	88
1	2	1	2	2	2	2	2	2
1	2	1	2	2	2	2	2	2
1	2	2	2	2	1	2	2	2
1	1	1	2	2	2	2	2	2
1	1	1	2	2	2	2	2	2
1	1	2	2	2	2	1	2	2

COMPGAO	COMPGAIT	COMPGAAS	COMPGAC	COMPGAD	COMPGASI	COMPGATI	COMPGAST	COMPGATC
2	1	2	2	2	2	2	2	2
2	2	2	2	2	2	2	2	2
1	2	1	2	2	2	2	2	2
88	88	88	88	88	88	88	88	88
2	2	2	1	2	2	2	2	2
88	88	88	88	88	88	88	88	88
2	2	2	2	2	2	2	2	2
2	2	2	2	2	2	2	1	2
2	2	2	2	2	2	2	2	2
2	2	2	2	2	2	2	2	2
88	88	88	88	88	88	88	88	88
2	2	2	2	2	2	2	2	2
2	2	2	2	2	2	2	2	2
2	2	2	2	2	2	2	1	2
88	88	88	88	88	88	88	88	88
2	1	2	2	2	2	2	2	2
2	2	2	2	2	2	2	2	2
2	1	2	2	2	2	2	2	2
2	2	2	2	2	2	2	2	2
88	88	88	88	88	88	88	88	88
88	88	88	88	88	88	88	88	88
2	2	2	2	2	2	2	2	2
2	1	2	2	2	2	2	2	2
88	88	88	88	88	88	88	88	88
2	1	2	2	2	2	2	1	2
88	88	88	88	88	88	88	88	88
88	88	88	88	88	88	88	88	88
1	2	2	2	2	2	2	2	2
2	2	2	2	2	2	2	2	2
2	2	1	2	2	2	2	2	2
2	1	2	2	2	2	2	2	2
2	1	2	2	2	2	2	1	2
2	2	2	2	2	2	2	2	2
88	88	88	88	88	88	88	88	88
2	1	2	2	2	2	2	2	2
88	88	88	88	88	88	88	88	88
2	2	2	2	2	2	2	2	2
2	2	2	2	2	2	2	2	2
88	88	88	88	88	88	88	88	88
2	1	2	2	2	2	2	2	2
88	88	88	88	88	88	88	88	88
88	88	88	88	88	88	88	88	88
88	88	88	88	88	88	88	88	88
2	2	2	2	2	2	2	1	2
2	2	2	2	2	2	2	2	2
2	1	2	2	2	2	2	2	2
88	88	88	88	88	88	88	88	88
2	2	2	2	2	2	2	2	2
88	88	88	88	88	88	88	88	88

2	2	2	2	2	2	2	1	2
2	2	2	2	2	2	2	2	2
2	1	2	2	2	2	2	2	2
88	88	88	88	88	88	88	88	88
88	88	88	88	88	88	88	88	88
2	2	2	2	2	2	2	2	2
2	1	2	2	2	2	2	2	2
2	1	2	2	2	2	2	2	2
2	1	2	1	2	2	2	2	2
2	2	2	2	2	2	2	2	2
88	88	88	88	88	88	88	88	88
2	2	2	2	2	2	2	2	2
2	1	2	2	2	2	2	2	2
88	88	88	88	88	88	88	88	88
2	2	2	2	2	2	2	1	2
88	88	88	88	88	88	88	88	88
2	1	2	2	2	2	2	2	2
88	88	88	88	88	88	88	88	88
2	2	2	2	2	2	2	2	1
2	1	2	2	2	2	2	2	2
88	88	88	88	88	88	88	88	88
88	88	88	88	88	88	88	88	88
2	1	2	2	2	2	2	2	2
2	1	2	1	2	2	2	2	2
88	88	88	88	88	88	88	88	88
2	2	2	2	2	2	2	2	2
1	2	2	2	2	2	2	2	2
88	88	88	88	88	88	88	88	88
2	2	2	2	2	2	2	2	2
2	2	2	2	2	2	2	2	2
2	2	2	2	1	2	2	2	2
2	2	2	2	2	2	2	2	2
2	2	2	2	2	2	2	2	2
88	88	88	88	88	88	88	88	88
88	88	88	88	88	88	88	88	88
88	88	88	88	88	88	88	88	88
2	2	2	1	2	2	2	2	2
2	2	2	2	2	2	2	2	2
2	2	2	2	2	2	2	2	2
2	1	2	2	2	2	2	2	2
88	88	88	88	88	88	88	88	88
88	88	88	88	88	88	88	88	88
2	2	2	2	2	2	2	1	2
2	2	2	2	2	2	2	2	2
2	2	2	2	2	2	2	2	2
88	88	88	88	88	88	88	88	88
88	88	88	88	88	88	88	88	88
2	1	2	2	2	2	2	2	2
88	88	88	88	88	88	88	88	88
2	2	2	2	2	2	2	2	2

88	88	88	88	88	88	88	88	88
2	2	1	2	2	2	2	2	2
88	88	88	88	88	88	88	88	88
2	2	2	2	2	2	2	1	2
88	88	88	88	88	88	88	88	88
88	88	88	88	88	88	88	88	88
2	1	2	2	2	2	2	2	2
2	2	2	2	2	2	2	2	2
2	2	2	1	2	2	2	2	2
1	1	2	2	2	2	2	2	2
88	88	88	88	88	88	88	88	88
2	2	2	2	2	2	2	2	2
88	88	88	88	88	88	88	88	88
88	88	88	88	88	88	88	88	88
88	88	88	88	88	88	88	88	88
2	1	2	2	2	2	2	2	2
2	2	2	2	2	2	2	1	2
2	2	2	2	2	2	2	1	2
88	88	88	88	88	88	88	88	88
2	1	2	2	2	2	2	2	2
88	88	88	88	88	88	88	88	88
2	2	2	2	2	2	2	2	2
88	88	88	88	88	88	88	88	88
88	88	88	88	88	88	88	88	88
2	2	2	2	2	2	2	1	2
2	2	2	2	2	2	2	2	2
88	88	88	88	88	88	88	88	88
2	1	2	2	2	2	2	2	2
2	2	2	2	2	2	2	2	2
2	1	2	2	2	2	2	2	2
2	2	1	2	2	2	2	2	2
2	2	2	2	1	2	2	2	2
2	2	2	2	2	2	2	2	2
1	2	1	2	2	2	2	2	2
2	2	2	2	1	2	2	2	2
88	88	88	88	88	88	88	88	88
88	88	88	88	88	88	88	88	88
88	88	88	88	88	88	88	88	88
2	2	2	2	2	2	2	1	1
2	2	2	2	2	2	2	2	2
2	1	2	2	2	2	2	2	2
88	88	88	88	88	88	88	88	88
2	2	2	1	2	2	2	2	2
2	1	2	2	2	2	2	2	2
88	88	88	88	88	88	88	88	88
2	2	2	2	2	2	2	2	2
88	88	88	88	88	88	88	88	88
2	2	1	2	2	2	2	2	2
2	2	2	2	1	2	2	1	2
88	88	88	88	88	88	88	88	88

2	1	2	2	2	2	2	2	2
88	88	88	88	88	88	88	88	88
2	1	2	2	2	2	2	2	2
2	2	2	2	2	2	2	2	2
2	2	2	2	2	2	2	2	2
2	1	2	1	2	2	2	2	2
2	2	2	2	2	2	2	2	2
88	88	88	88	88	88	88	88	88
2	2	2	2	2	2	2	2	2
2	2	2	2	2	2	2	2	2
2	2	2	2	2	2	2	2	2
2	2	2	2	2	2	2	2	2
2	2	2	2	2	2	1	2	2
2	2	2	2	2	2	2	1	2
88	88	88	88	88	88	88	88	88
2	1	2	2	2	2	2	2	2
2	2	2	2	2	2	2	2	2
88	88	88	88	88	88	88	88	88
88	88	88	88	88	88	88	88	88
1	1	2	2	2	2	2	2	2
88	88	88	88	88	88	88	88	88
88	88	88	88	88	88	88	88	88
88	88	88	88	88	88	88	88	88
88	88	88	88	88	88	88	88	88
2	1	2	2	2	2	2	2	2
2	2	1	2	2	2	2	2	2
88	88	88	88	88	88	88	88	88
2	2	2	2	2	2	2	2	2
2	1	2	2	2	2	2	1	2
2	2	2	2	2	2	2	1	2
2	2	2	2	2	2	2	2	2
2	2	2	2	2	2	2	1	2
2	2	2	2	2	2	2	2	2
2	2	1	2	2	2	2	2	2
2	1	2	2	2	2	2	1	2
88	88	88	88	88	88	88	88	88
88	88	88	88	88	88	88	88	88
88	88	88	88	88	88	88	88	88
2	1	2	2	2	2	2	2	2
2	2	2	2	2	2	2	2	2
88	88	88	88	88	88	88	88	88
88	88	88	88	88	88	88	88	88
2	2	2	2	2	2	2	1	2
2	2	2	2	2	2	2	2	1
2	2	1	2	2	2	2	2	2
88	88	88	88	88	88	88	88	88
88	88	88	88	88	88	88	88	88
2	1	2	2	2	2	2	2	2
2	1	2	2	2	2	2	2	2
88	88	88	88	88	88	88	88	88

88	88	88	88	88	88	88	88	88
88	88	88	88	88	88	88	88	88
2	2	2	2	2	2	2	1	2
88	88	88	88	88	88	88	88	88
1	2	2	2	2	2	2	2	2
2	1	2	2	2	2	2	2	2
2	2	2	1	2	2	2	2	2
2	1	2	2	2	2	2	2	2
2	2	2	2	2	2	2	1	2
88	88	88	88	88	88	88	88	88
88	88	88	88	88	88	88	88	88
2	2	2	2	2	2	2	1	2
2	2	2	2	2	2	2	2	2
2	1	2	2	2	2	2	2	2
88	88	88	88	88	88	88	88	88
1	1	2	2	2	2	2	2	2
2	2	2	2	2	2	2	2	2
2	1	2	2	2	2	2	2	2
2	1	2	2	2	2	2	2	2
2	2	2	2	2	2	2	1	2
88	88	88	88	88	88	88	88	88
2	2	2	2	2	2	2	2	2
1	2	2	2	2	2	2	1	2
2	2	2	2	2	2	2	2	2
2	1	2	2	2	2	2	2	2
88	88	88	88	88	88	88	88	88
2	2	2	2	2	2	2	1	2
2	2	2	2	2	2	2	2	2
1	2	2	2	2	2	2	2	2
88	88	88	88	88	88	88	88	88
2	2	1	2	2	2	2	2	2
2	2	2	2	2	2	2	2	2
2	2	2	2	2	2	2	1	2
2	1	1	2	2	2	2	2	2
88	88	88	88	88	88	88	88	88
2	2	2	2	2	2	2	2	2
88	88	88	88	88	88	88	88	88
2	2	1	2	2	2	2	2	2
2	2	2	2	1	2	2	2	2
88	88	88	88	88	88	88	88	88
2	1	1	1	2	2	2	2	2
1	1	2	2	2	2	2	2	2
2	2	2	2	2	2	2	2	2
88	88	88	88	88	88	88	88	88
1	2	2	2	2	2	2	2	2
88	88	88	88	88	88	88	88	88
2	1	2	2	2	2	2	1	2
88	88	88	88	88	88	88	88	88
1	2	2	2	2	2	2	2	2
88	88	88	88	88	88	88	88	88

88	88	88	88	88	88	88	88	88
1	2	2	2	2	2	2	1	2
88	88	88	88	88	88	88	88	88
2	2	2	2	2	2	2	2	2
2	2	1	2	2	2	2	2	2
2	2	2	2	2	2	2	2	2
88	88	88	88	88	88	88	88	88
2	2	1	2	2	2	2	2	2
2	2	2	2	1	2	2	2	2
2	2	2	2	2	1	2	2	2
88	88	88	88	88	88	88	88	88
2	2	2	2	2	2	2	2	1
1	1	1	2	2	2	2	2	2
2	2	2	2	2	2	2	2	2
2	2	2	2	2	2	2	2	1
2	2	2	2	2	2	2	2	2
2	1	1	2	2	2	2	2	2
2	2	2	2	2	2	2	2	2
2	2	2	2	2	2	2	2	2
2	1	2	2	2	2	2	2	2
2	2	2	2	2	2	2	2	2
88	88	88	88	88	88	88	88	88
88	88	88	88	88	88	88	88	88
88	88	88	88	88	88	88	88	88
2	2	1	2	2	2	2	2	2
1	1	2	2	2	2	2	2	2
88	88	88	88	88	88	88	88	88
2	2	2	2	2	2	2	2	2
2	1	2	2	2	2	2	2	2
2	1	1	2	2	2	2	2	2
2	1	2	2	2	2	2	2	2
2	2	2	2	2	2	2	2	2
2	2	2	2	2	2	2	2	2

COMPGAÚ	COMPGABI	COMPGAC	COMPGAH	COMPGACI	COMPGAV	COMPGAES	COMPGAM	COMPGATV
2	2	2	2	2	2	2	2	2
2	2	2	2	2	2	2	2	2
2	2	2	2	2	2	2	2	2
88	88	88	88	88	88	88	88	88
2	2	2	2	2	2	2	2	2
88	88	88	88	88	88	88	88	88
2	2	2	2	2	2	2	2	2
2	2	2	2	2	2	2	2	2
2	2	2	2	2	2	2	2	2
2	2	2	2	2	2	2	2	2
88	88	88	88	88	88	88	88	88
2	2	2	2	2	2	2	2	2
2	2	2	2	2	2	2	2	2
2	2	2	2	2	2	2	2	2
88	88	88	88	88	88	88	88	88
2	2	2	2	2	2	2	2	2
2	2	2	2	2	2	2	2	2
2	2	2	2	2	2	2	2	2
2	2	2	2	1	2	2	2	2
88	88	88	88	88	88	88	88	88
88	88	88	88	88	88	88	88	88
2	2	2	2	2	2	2	2	2
2	2	2	2	2	2	2	2	2
88	88	88	88	88	88	88	88	88
2	2	2	2	2	2	2	2	2
88	88	88	88	88	88	88	88	88
88	88	88	88	88	88	88	88	88
2	2	2	2	2	2	2	2	2
2	2	2	2	2	2	2	2	2
2	2	2	2	2	2	2	2	2
2	2	2	2	2	2	2	2	2
2	2	2	2	2	2	2	2	2
2	2	2	2	2	1	2	2	2
88	88	88	88	88	88	88	88	88
2	2	2	2	2	2	2	2	2
88	88	88	88	88	88	88	88	88
2	2	2	2	2	2	1	2	2
2	2	2	2	2	2	2	2	2
88	88	88	88	88	88	88	88	88
2	2	2	2	2	2	2	2	2
88	88	88	88	88	88	88	88	88
88	88	88	88	88	88	88	88	88
88	88	88	88	88	88	88	88	88
2	2	2	2	2	2	2	2	2
2	2	2	2	2	2	2	2	2
2	2	2	2	2	2	2	2	2
88	88	88	88	88	88	88	88	88
2	2	2	2	2	2	2	2	2
88	88	88	88	88	88	88	88	88

2	2	2	2	2	2	2	1	2
2	2	2	2	2	2	2	2	2
2	2	2	2	2	2	2	2	2
88	88	88	88	88	88	88	88	88
88	88	88	88	88	88	88	88	88
2	2	2	2	2	1	2	2	2
2	2	1	2	2	2	2	2	2
2	2	2	2	2	2	2	2	2
2	2	2	2	2	2	2	2	2
2	2	2	2	2	2	2	2	2
88	88	88	88	88	88	88	88	88
2	2	2	2	2	2	2	2	2
2	2	2	2	2	2	2	2	2
88	88	88	88	88	88	88	88	88
2	2	2	2	2	2	2	2	2
88	88	88	88	88	88	88	88	88
2	2	2	2	2	2	2	2	2
88	88	88	88	88	88	88	88	88
2	2	2	2	2	2	2	2	2
2	2	2	2	2	2	2	2	2
88	88	88	88	88	88	88	88	88
88	88	88	88	88	88	88	88	88
2	2	2	2	2	2	2	2	2
2	2	2	2	2	2	2	2	2
88	88	88	88	88	88	88	88	88
2	2	2	2	2	2	2	2	2
2	2	2	2	2	2	2	2	2
88	88	88	88	88	88	88	88	88
2	2	2	2	2	2	2	2	2
2	2	2	2	2	2	2	2	2
2	2	2	2	2	2	2	2	2
2	2	2	2	2	2	2	2	2
2	2	2	2	2	2	2	2	2
88	88	88	88	88	88	88	88	88
88	88	88	88	88	88	88	88	88
88	88	88	88	88	88	88	88	88
2	2	2	2	2	2	2	2	2
2	2	2	2	2	2	2	2	2
2	2	2	2	2	2	2	2	2
2	2	1	2	2	2	2	2	2
88	88	88	88	88	88	88	88	88
88	88	88	88	88	88	88	88	88
2	2	2	2	2	2	2	2	2
2	2	2	2	2	2	2	2	2
2	2	2	2	2	2	2	2	2
88	88	88	88	88	88	88	88	88
88	88	88	88	88	88	88	88	88
2	2	2	2	2	2	2	2	2
88	88	88	88	88	88	88	88	88
2	2	2	2	2	2	2	2	2

88	88	88	88	88	88	88	88	88
2	2	2	2	2	2	2	2	2
88	88	88	88	88	88	88	88	88
2	2	2	2	2	2	2	2	2
88	88	88	88	88	88	88	88	88
88	88	88	88	88	88	88	88	88
2	2	2	2	2	2	2	2	2
2	2	2	2	2	2	2	2	2
2	2	2	2	2	2	2	2	2
2	2	2	2	2	2	2	2	2
88	88	88	88	88	88	88	88	88
2	2	2	2	2	2	2	2	2
88	88	88	88	88	88	88	88	88
88	88	88	88	88	88	88	88	88
88	88	88	88	88	88	88	88	88
2	2	2	2	2	2	2	2	2
2	2	2	2	2	2	2	2	2
2	2	2	2	2	2	2	2	2
88	88	88	88	88	88	88	88	88
2	2	2	2	2	2	2	2	2
88	88	88	88	88	88	88	88	88
2	2	2	2	2	2	2	2	2
88	88	88	88	88	88	88	88	88
88	88	88	88	88	88	88	88	88
2	2	2	2	2	2	2	2	2
2	2	2	2	2	2	2	2	2
88	88	88	88	88	88	88	88	88
2	2	2	2	2	2	2	2	2
2	2	2	2	2	2	2	2	2
2	2	2	2	2	2	2	2	2
2	2	2	2	2	2	2	2	2
2	2	2	2	2	1	2	2	2
2	2	2	2	2	2	2	2	2
2	2	2	2	2	2	2	2	2
2	2	2	2	2	2	2	2	2
88	88	88	88	88	88	88	88	88
88	88	88	88	88	88	88	88	88
88	88	88	88	88	88	88	88	88
2	2	2	2	2	2	2	2	2
2	2	2	2	2	2	2	2	2
2	2	2	2	2	2	2	2	2
88	88	88	88	88	88	88	88	88
2	2	2	2	2	2	2	2	2
2	2	2	2	2	2	2	2	2
88	88	88	88	88	88	88	88	88
2	2	2	2	2	2	2	2	2
88	88	88	88	88	88	88	88	88
2	2	2	2	2	2	2	2	2
2	2	2	2	2	2	2	2	2
88	88	88	88	88	88	88	88	88

2	2	2	2	2	2	2	2	2
88	88	88	88	88	88	88	88	88
2	2	2	2	2	2	2	2	2
2	2	2	2	2	2	2	2	2
2	2	2	2	2	2	2	2	2
2	2	2	2	2	2	2	2	2
2	2	2	2	2	2	2	2	2
88	88	88	88	88	88	88	88	88
2	2	2	2	2	2	2	2	2
2	2	2	2	2	2	2	2	2
2	2	2	2	2	2	2	2	2
2	2	2	2	2	2	2	2	2
2	2	2	2	2	2	2	2	2
2	2	2	2	2	2	2	2	2
88	88	88	88	88	88	88	88	88
2	2	2	2	2	2	2	2	2
2	2	2	2	2	2	2	2	2
88	88	88	88	88	88	88	88	88
88	88	88	88	88	88	88	88	88
2	1	2	2	2	2	2	2	2
88	88	88	88	88	88	88	88	88
88	88	88	88	88	88	88	88	88
88	88	88	88	88	88	88	88	88
88	88	88	88	88	88	88	88	88
2	2	2	2	2	2	2	2	2
2	2	2	2	2	2	2	2	2
88	88	88	88	88	88	88	88	88
2	2	2	2	2	2	2	2	2
2	2	2	2	2	2	2	2	2
2	2	2	2	2	2	2	2	2
2	2	2	2	2	2	2	2	2
2	2	2	2	2	2	2	2	2
2	2	1	2	2	2	2	2	2
2	2	2	2	2	2	2	2	2
2	2	2	2	2	2	2	2	2
88	88	88	88	88	88	88	88	88
88	88	88	88	88	88	88	88	88
88	88	88	88	88	88	88	88	88
2	2	2	2	2	2	2	2	2
2	2	2	2	2	2	2	2	2
88	88	88	88	88	88	88	88	88
88	88	88	88	88	88	88	88	88
2	2	2	2	2	2	2	2	2
2	2	2	2	2	2	2	2	2
2	2	2	2	2	2	2	2	2
88	88	88	88	88	88	88	88	88
88	88	88	88	88	88	88	88	88
2	2	2	2	2	2	2	2	2
2	2	2	2	2	2	2	2	2
88	88	88	88	88	88	88	88	88

88	88	88	88	88	88	88	88	88
88	88	88	88	88	88	88	88	88
2	2	2	2	2	2	2	2	2
88	88	88	88	88	88	88	88	88
2	2	2	2	2	2	2	2	2
2	2	2	2	2	2	2	2	2
2	2	2	2	2	2	2	2	2
2	2	2	2	2	2	2	2	2
2	2	2	2	2	2	2	2	2
88	88	88	88	88	88	88	88	88
88	88	88	88	88	88	88	88	88
2	2	2	2	2	2	2	2	2
2	2	2	2	2	2	2	2	2
2	2	2	2	2	2	2	2	2
88	88	88	88	88	88	88	88	88
2	2	2	2	2	2	2	2	2
2	2	2	2	2	2	2	2	2
2	2	2	2	2	2	2	2	2
2	2	2	2	2	2	2	2	2
2	2	1	2	2	2	2	2	2
88	88	88	88	88	88	88	88	88
2	2	2	2	2	2	2	2	2
2	2	2	2	2	2	2	2	2
2	2	2	2	2	2	2	2	2
2	2	2	2	2	2	2	2	2
88	88	88	88	88	88	88	88	88
2	2	2	2	2	2	2	2	2
2	2	2	2	2	2	2	2	1
2	2	2	2	2	2	2	2	2
88	88	88	88	88	88	88	88	88
2	2	2	2	2	2	2	2	2
2	2	2	2	2	2	2	2	22
2	2	2	2	2	2	2	2	2
2	2	2	2	2	2	2	2	2
88	88	88	88	88	88	88	88	88
2	2	2	2	2	2	2	2	2
88	88	88	88	88	88	88	88	88
2	2	2	2	2	2	2	2	2
2	2	2	2	2	2	2	2	2
88	88	88	88	88	88	88	88	88
2	2	2	2	2	2	2	2	2
2	2	2	2	2	2	2	2	2
2	2	2	2	2	2	2	2	2
88	88	88	88	88	88	88	88	88
2	2	2	2	2	2	2	2	2
88	88	88	88	88	88	88	88	88
2	2	2	2	2	2	2	2	2
88	88	88	88	88	88	88	88	88
2	2	1	2	2	2	2	2	2
88	88	88	88	88	88	88	88	88

88	88	88	88	88	88	88	88	88
2	2	2	2	2	2	2	2	2
88	88	88	88	88	88	88	88	88
2	2	2	2	2	2	2	2	2
2	2	2	2	2	2	2	2	2
2	2	2	2	2	2	2	2	2
88	88	88	88	88	88	88	88	88
2	2	2	2	2	2	2	2	2
2	2	2	2	2	2	2	2	2
2	2	2	2	2	2	2	2	2
88	88	88	88	88	88	88	88	88
2	2	2	2	2	2	2	2	2
2	2	2	2	2	2	2	2	2
1	2	2	2	2	2	2	2	2
2	2	2	2	2	2	2	2	2
2	2	2	2	2	2	2	2	2
2	2	2	2	2	2	2	2	2
2	2	2	2	2	2	2	2	2
2	2	2	2	2	2	2	2	2
2	2	2	2	2	2	2	2	2
2	2	2	2	2	2	2	2	2
88	88	88	88	88	88	88	88	88
88	88	88	88	88	88	88	88	88
88	88	88	88	88	88	88	88	88
2	2	2	2	2	2	2	2	2
2	2	2	2	2	2	2	2	2
88	88	88	88	88	88	88	88	88
2	2	2	2	2	2	2	2	2
2	2	2	2	2	2	2	2	2
2	2	2	2	2	2	2	2	2
2	2	2	2	2	2	2	2	2
2	2	2	1	2	2	2	2	2
2	2	2	2	2	2	2	2	2

COMPGAC/	COMPGAPI	TABAG	TABAGCIG	MOTIVOIN'	GESTA	PARA	CESÁREA	ABORTO
2	2	2	88	Assistência	3	2	0	0
2	2	1	5	Assistência	1	0	0	0
2	2	2	88	Indução po	2	1	0	0
88	88	1	66	Aminiorrex	5	3	0	1
2	2	2	88	Trabalho d	2	1	0	0
88	88	2	88	Assistência	2	1	0	0
2	2	2	88	Trabalho d	5	3	0	1
2	2	2	88	Trabalho d	2	1	0	0
2	2	2	88	Interrupçã	2	1	0	0
2	2	2	88	Aminiorrex	2	1	0	0
88	88	2	88	Trabalho d	3	1	0	1
2	2	2	88	Interrupçã	2	1	0	0
2	2	2	88	Assistência	2	0	0	1
2	2	2	88	Assistência	3	2	0	0
88	88	2	88	Assistência	4	2	0	1
2	2	2	88	Amniorrexi	1	0	0	0
2	2	2	88	Assistência	2	1	0	0
2	2	2	88	Assistência	2	1	0	0
2	2	2	88	Assistência	3	1	0	1
88	88	2	88	Indução po	1	0	0	0
88	88	1	6	Trabalho d	3	2	0	0
2	2	2	88	Assistência	1	0	0	0
2	2	2	88	Aminiorrex	1	0	0	0
88	88	2	88	Assistência	3	2	0	0
2	2	1	66	Indução - C	1	0	0	0
88	88	2	88	Assistência	9	5	0	3
88	88	2	88	Trabalho d	2	0	0	1
2	2	2	88	Assistência	3	2	0	0
2	2	2	88	Trabalho d	2	1	0	0
2	2	1	66	Trabalho d	1	0	0	0
2	2	2	88	Assistência	3	2	0	0
2	2	2	88	Assistência	3	2	0	0
2	2	2	88	Amniorrexi	1	0	0	0
88	88	2	88	Assistência	2	0	1	0
2	2	2	88	Assistência	1	0	0	0
88	88	1	7	Assistência	6	2	0	2
2	2	2	88	Indução po	3	0	0	2
2	2	1	10	Amniorrexi	4	2	1	0
88	88	2	88	Aminiorrex	3	2	0	0
2	2	1	10	Assistência	1	0	0	0
88	88	1	2	Trabalho d	1	0	0	0
88	88	2	88	Indução ao	2	1	0	0
88	88	2	88	Assistência	4	3	0	0
2	2	2	88	Assistência	3	2	0	0
2	2	2	88	Assistência	1	0	0	0
2	2	2	88	Interrupçã	2	0	0	1
88	88	2	88	Assistência	3	2	0	0
2	2	2	88	Indução ao	3	1	0	1
88	88	2	88	Assistência	3	2	0	0

2	2	2	88	Interrupçã	1	0	0	0
2	2	2	88	Pré- eclâm	2	0	0	1
2	2	2	88	Trabaho de	3	2	0	0
88	88	2	88	Assistência	1	0	0	0
88	88	1	20	Assistência	1	0	0	0
2	2	2	88	Amniorrexi	2	1	0	0
2	2	2	88	Interna por	1	0	0	0
2	2	2	88	Assistência	1	0	0	0
2	2	2	88	Trabalho d	2	1	0	0
2	2	1	66	Assistência	2	1	0	0
88	88	2	88	Assistência	1	0	0	0
2	2	2	88	Assistência	5	4	0	0
2	2	2	88	Trabalho d	1	0	0	0
88	88	2	88	Assistência	1	0	0	0
2	2	2	88	Trabalho d	1	0	0	0
88	88	2	88	Trabalho d	3	1	0	1
2	2	2	88	Assistência	1	0	0	0
88	88	2	88	Assitência a	2	1	0	0
2	2	2	88	Indução po	1	0	0	0
2	2	1	66	Assistência	3	2	0	0
88	88	2	88	Trabalho d	4	2	0	1
88	88	2	88	Trabalho d	2	1	0	0
2	2	2	88	Trabalho d	2	1	0	0
2	2	2	88	Amniorrexi	2	1	0	0
88	88	2	88	Amniorrexi	2	1	0	0
2	2	2	88	Aminiorrex	1	0	0	0
2	2	2	88	Assistência	2	1	0	0
88	88	1	40	Trabalho d	6	5	0	0
2	2	1	5	Assistência	7	2	1	3
2	2	2	88	Trabalho d	1	0	0	0
2	2	2	88	Trabalho d	3	1	0	1
2	2	2	88	Trabalho d	3	1	0	1
2	2	2	88	Assistência	2	1	0	0
88	88	2	88	Indução ao	2	1	0	0
88	88	1	10	Trabalho d	1	0	0	0
88	88	2	88	Trabalho d	4	2	1	0
2	2	2	88	Trabalho d	1	0	0	0
2	2	1	66	Assistência	4	3	0	0
2	2	1	66	Assistência	4	2	0	1
2	2	2	88	Indução ao	2	1	0	0
88	88	2	88	Trabalho d	1	0	0	0
88	88	2	88	Trabalho d	3	1	0	1
2	2	2	88	Amniorrexi	1	0	0	0
2	2	2	88	Trabalho d	1	0	0	0
2	2	2	88	Trabalho d	4	0	0	3
88	88	2	88	Aminiorrex	2	1	0	0
88	88	2	88	Trabalho d	4	1	0	2
2	2	1	66	Trabalho d	1	0	0	0
88	88	2	88	Amniorrexi	1	0	0	0
1	2	2	88	Assistência	2	1	0	0

88	88	2	88	Assistência	3	2	0	0
2	2	2	88	Amniorrexi	1	0	0	0
88	88	2	88	Trabalho d	2	1	0	0
2	2	2	88	Amniorrexi	2	1	0	0
88	88	2	88	Início de T	2	1	0	0
88	88	2	88	Trabalho d	3	2	0	0
2	2	2	88	Suspeita de	6	4	0	1
2	2	2	88	Trabalho d	2	0	0	1
2	2	1	4	Assistência	4	3	0	0
2	2	2	88	Trabalho d	2	1	0	0
88	88	1	66	Trabalho d	4	3	0	0
2	2	2	88	Amniorrexi	1	0	0	0
88	88	1	6	Assistência	2	1	0	0
88	88	1	10	Trabalho d	3	2	0	0
88	88	2	88	Assistência	2	1	0	0
2	2	2	88	Indução ao	3	1	1	0
2	2	2	88	Indução ao	2	1	0	0
2	2	1	20	Assistência	3	2	0	0
88	88	2	88	Trabalho d	3	2	0	0
2	2	2	88	Trabalho d	5	2	0	2
88	88	2	88	Trabalho d	2	0	1	0
2	2	1	15	Indução po	3	2	0	0
88	88	2	88	Indução ao	1	0	0	0
88	88	2	88	Trabalho d	1	0	0	0
2	2	2	88	Indução ao	2	1	0	0
2	2	2	88	Trabalho d	1	0	0	0
88	88	2	88	Assistência	3	1	0	1
2	2	1	15	Assistência	4	3	0	0
2	2	2	88	Assistência	5	3	1	0
2	2	1	66	Indução ao	1	0	0	0
2	2	2	88	Assistência	2	0	0	1
2	2	1	66	Assistência	1	0	0	0
2	2	2	88	Amniorrexi	3	2	0	0
2	2	1	2	Assistência	7	6	0	0
2	2	2	88	Indução ao	1	0	0	0
88	88	2	88	Amniorrexi	1	0	0	0
88	88	1	10	Assistência	5	4	0	0
88	88	2	88	Amniorrexi	1	0	0	0
2	2	2	88	Trabalho d	1	0	0	0
2	2	1	66	Assistência	2	1	0	0
2	2	2	88	Assistência	1	0	0	0
88	88	2	88	Assistência	3	2	0	0
2	2	2	88	Trabalho d	1	0	0	0
2	2	2	88	Indução ao	3	2	0	0
88	88	2	88	Aminiorrex	1	0	0	0
2	2	2	88	Assistência	4	3	0	0
88	88	2	88	Trabalho d	2	1	0	0
2	2	2	88	Assistência	2	1	0	0
2	2	1	2	Assistência	3	1	1	0
88	88	1	10	Assistência	5	4	0	0

2	2	2	88	Inducação a	1	0	0	0
88	88	2	88	Assistência	2	1	0	0
2	2	2	88	Aminiorrex	8	7	0	0
2	2	2	88	Pós-datism	1	0	0	0
2	2	2	88	Trabalho de	1	0	0	0
2	2	2	88	Aminiorrex	4	1	0	1
2	2	2	88	Encaminha	1	0	0	0
88	88	2	88	Trabalho de	4	2	0	1
2	2	2	88	Aminiorrex	1	0	0	0
2	2	2	88	Aminiorrex	2	1	0	0
2	2	2	88	Trabalho de	2	1	0	0
2	2	2	88	Trabalho de	5	2	0	2
2	2	1	66	Trabalho de	4	3	0	0
2	2	2	88	Aminiorrex	2	1	0	0
88	88	1	66	Trabalho de	3	1	0	1
2	2	1	10	Trabalho de	2	1	0	0
2	2	2	88	Indução po	2	0	0	1
88	88	2	88	Amniorrexi	1	0	0	0
88	88	2	88	Indução do	3	1	1	0
2	2	2	88	Aminiorrex	1	0	0	0
88	88	1	66	Trabalho de	1	0	0	0
88	88	2	88	Assistência	1	0	0	0
88	88	2	88	Indução TP	7	3	0	2
88	88	1	5	Trabalho de	3	1	1	0
2	2	2	88	Assistência	1	0	0	0
2	2	2	88	Indução po	1	0	0	0
88	88	2	88	Trabalho de	1	0	0	0
2	2	2	88	Pré-eclâmp	1	0	0	0
2	2	1	66	Trabalho de	2	1	0	0
2	2	2	88	Trabalho de	1	0	0	0
2	2	2	88	Trabalho de	2	1	0	0
2	2	2	88	Indução ao	1	0	0	0
2	2	2	88	Colestase g	5	4	0	0
2	2	2	88	Trabalho de	1	0	0	0
2	2	2	88	Indução ao	3	2	0	0
88	88	2	88	Indução ao	2	1	0	0
88	88	2	88	Trabalho de	3	2	0	0
88	88	2	88	Assistência	3	1	0	1
2	2	2	88	Trabalho de	3	0	0	2
2	2	2	88	TA elevada	1	0	0	0
88	88	2	88	Indução ao	1	0	0	0
88	88	2	88	Assistência	1	0	0	0
2	2	2	88	Trabalho de	2	1	0	0
2	2	2	88	Interrupção	2	0	1	0
2	2	2	88	Pré-eclâmp	4	0	3	0
88	88	2	88	Aminiorrex	1	0	0	0
88	88	2	88	Por contraç	3	0	2	0
2	2	2	88	Interrupção	3	1	1	0
2	2	2	88	Cesaréa po	2	0	1	0
88	88	2	88	Amniorrexi	2	0	1	0

88	88	2	88	Cesariana -	4	3	0	0
88	88	2	88	Aminiorrex	1	0	0	0
2	2	2	88	Indução ao	1	0	0	0
88	88	2	88	Cesariana -	1	0	0	0
2	2	2	88	Interrupçã	4	0	2	1
2	2	1	88	Amniorrexi	1	0	0	0
2	2	2	88	Interrupçã	7	3	3	0
2	2	2	88	Interrupçã	4	0	3	0
2	2	2	88	Indução ao	1	0	0	0
88	88	2	88	Indução ao	1	0	0	0
88	88	2	88	Interrupçã	3	0	1	1
2	2	1	66	Interrupçã	5	0	2	2
2	2	2	88	Amniorrexi	1	0	0	0
2	2	2	88	Aminiorrex	1	0	0	0
88	88	2	88	Assistência	2	0	0	1
2	2	2	88	Aumento d	1	0	0	0
2	2	2	88	Interrupçã	2	0	1	0
2	2	2	88	Indução ao	1	0	0	0
2	2	2	88	Indução ao	1	0	0	0
2	2	1	4	Indução do	1	0	0	0
88	88	2	88	Cesárea ele	3	1	1	0
2	2	2	88	Indução ao	1	0	0	0
2	2	2	88	Controle TA	4	2	0	1
2	2	2	88	Cesárea ele	2	0	1	0
2	2	1	3	Indução ao	1	0	0	0
88	88	2	88	Amniorrexi	1	0	0	0
2	2	1	10	Amniorrexi	1	0	0	0
2	2	2	88	Vem para a	1	0	0	0
2	2	2	88	Interrupçã	3	0	1	1
88	88	88	88	88	88	88	88	88
2	2	2	88	Interrupçã	2	0	1	0
2	2	2	88	Indução do	1	0	0	0
2	2	2	88	Assistência	1	0	0	0
2	2	1	5	Interna par	6	1	0	4
88	88	1	66	Interrupçã	5	3	1	0
2	1	2	88	Indução ao	2	1	0	0
88	88	2	88	Assistência	1	0	0	0
2	2	1	9	Indução ao	2	0	0	1
2	2	2	88	Amniorrexi	1	0	0	0
88	88	2	88	Indução ao	1	0	0	0
2	2	1	2	Trabalho d	2	1	0	0
2	2	2	88	Interrupçã	3	0	1	1
2	2	2	88	Trabalho d	1	0	0	0
88	88	2	88	Trabalho d	2	0	1	0
2	2	1	66	Assistência	1	0	0	0
88	88	2	88	Amniorrexi	1	0	0	0
2	2	2	88	Interrupçã	2	0	1	0
88	88	88	88	88	88	88	88	88
2	2	2	88	Prurido ges	1	0	0	0
88	88	88	88	88	88	88	88	88

88	88	2	88	Trabalho d	3	0	2	0
2	2	1	5	Planejamer	2	1	0	0
88	88	2	88	Trabalho d	2	0	1	0
2	2	2	88	Interrupçã	4	2	1	0
2	2	2	88	Indução do	1	0	0	0
2	2	2	88	Não entrou	3	1	0	1
88	88	88	88	88	88	88	88	88
2	2	2	88	Indução do	1	0	0	0
2	2	1	66	Trabalho d	1	0	0	0
2	2	2	88	Aminiorrex	3	1	1	0
88	88	88	88	88	88	88	88	88
2	2	2	88	Aminiorrex	1	0	0	0
2	2	1	40	Suspeita de	2	0	1	0
2	2	2	88	Cesariana p	2	0	1	0
2	2	2	88	Pré-eclâmp	1	0	0	0
2	2	2	88	Trabalho d	3	0	0	2
2	2	2	88	Trabalho d	1	0	0	0
2	2	1	66	Interna por	1	0	0	0
2	2	1	5	Interrupçã	3	2	0	1
2	2	1	66	Assistência	3	1	1	0
2	2	2	88	Trabalho d	3	0	0	2
88	88	2	88	Aminiorrex	4	2	0	1
88	88	2	88	Assistência	3	1	0	0
88	88	2	88	Por 3 cesár	4	0	3	0
2	2	2	88	Trabalho d	2	0	0	0
2	2	2	88	Heparina p	6	3	2	0
88	88	1	66	Indução ao	2	1	0	0
2	2	2	88	Cesárea inc	3	0	2	0
2	2	2	88	Cesariana p	2	0	1	0
2	2	2	88	Rupreme -	1	0	0	0
2	2	1	66	Cesariana p	2	0	1	0
2	2	2	88	Indução do	1	0	0	0
2	2	1	6	Interrupçã	7	2	2	2

ECTÓPICA	COMPGP	COMPGQU	TIPARTO	USOFOR	LAC	GRAULAC	SUTLAC	EPISIO
0	2	88	1	2	2	88	88	2
0	88	88	1	2	2	88	88	1
0	2	88	1	2	2	88	88	2
0	2	88	1	2	2	88	88	2
0	2	88	1	2	2	88	88	2
0	2	88	1	2	1	3	1	2
0	2	88	1	2	1	2	1	2
0	1	PE	1	2	2	88	88	1
0	2	88	1	2	2	88	88	2
0	1	HPP	1	2	1	2	1	2
0	2	88	1	2	2	88	88	2
0	2	88	1	2	2	88	88	2
0	1	aborto	1	2	2	88	88	1
0	2	88	1	2	1	1	1	2
0	2	88	1	2	2	88	88	2
0	88	88	1	2	1	2	1	2
0	2	88	1	2	2	88	88	2
0	2	88	1	2	1	2	1	2
0	1	ABORTO - (	1	2	2	88	88	2
0	88	88	1	2	2	88	88	1
0	2	88	1	2	2	88	88	2
0	88	88	1	2	1	1	1	2
0	88	88	1	2	2	88	88	1
0	2	88	1	2	2	88	88	2
0	88	88	1	2	1	2	1	2
0	1	HPP	1	2	1	1	1	2
0	1	aborto	1	2	2	88	88	1
0	2	88	1	2	2	88	88	2
0	2	88	1	2	1	2	1	2
0	88	88	1	2	2	88	88	1
0	2	88	1	2	1	2	1	2
0	2	88	1	2	1	1	1	2
0	88	88	1	2	2	88	88	1
0	2	88	1	2	2	88	88	1
0	88	88	1	1	1	66	1	1
1	1	Gestação e	1	2	2	88	88	2
0	1	Insuficiênci	1	2	1	1	1	2
0	1	FETO MOR	1	1	2	88	88	1
0	2	88	1	2	1	2	1	2
0	88	88	1	2	1	66	1	1
0	88	88	1	1	2	88	88	1
0	2	88	1	2	1	2	1	2
0	2	88	1	2	2	88	88	2
0	2	88	1	2	1	1	1	2
0	88	88	1	2	2	88	88	2
0	1	CURETAGE	1	2	1	2	1	1
0	2	88	1	2	1	1	1	2
0	2	88	1	2	1	2	1	2
0	2	88	1	2	1	1	1	2

0	88	88	1	2	2	88	88	1
0	2	88	1	2	1	1	1	2
0	2	88	1	2	1	1	1	2
0	88	88	1	2	2	88	88	1
0	88	88	1	2	2	88	88	1
0	2	88	1	2	2	88	88	2
0	88	88	1	2	1	1	1	2
0	88	88	1	2	2	88	88	2
0	2	88	1	2	1	1	1	2
0	2	88	1	2	2	88	88	1
0	88	88	1	2	1	2	1	2
0	2	88	1	2	2	88	88	2
0	88	88	1	1	2	88	88	1
0	88	88	1	2	1	1	1	2
0	88	88	1	2	2	88	88	1
0	2	88	1	2	2	88	88	1
0	88	88	1	2	2	88	88	1
0	2	88	1	2	1	1	2	2
0	88	88	1	2	1	1	1	2
0	2	88	1	2	2	88	88	2
0	2	88	1	2	2	88	88	1
0	2	88	1	2	1	2	1	2
0	1	HPP	1	2	1	1	1	2
0	2	88	1	2	1	1	1	2
0	2	88	1	2	2	88	88	2
0	88	88	1	1	2	88	88	2
0	2	88	1	2	2	88	88	1
0	2	88	1	2	2	88	88	2
0	2	88	1	2	1	1	2	2
0	88	88	1	1	2	88	88	1
0	2	88	1	2	1	1	1	2
0	2	88	1	2	1	1	1	2
0	2	88	1	2	2	88	88	2
0	2	88	1	2	1	1	1	2
0	88	88	1	2	1	2	1	2
0	1	Eclâmpsia	1	2	2	88	88	2
0	88	88	1	2	2	88	88	2
0	2	88	1	2	2	88	88	2
0	1	Corioamnic	1	2	2	88	88	2
0	2	88	1	2	1	1	1	2
0	88	88	1	2	2	88	88	1
0	2	88	1	2	2	88	88	1
0	88	88	1	1	2	88	88	1
0	88	88	1	2	1	1	1	1
0	1	aborto	1	1	1	3	1	2
0	2	88	1	2	1	1	1	2
0	2	88	1	2	2	88	88	1
0	88	88	1	1	2	88	88	1
0	88	88	1	1	2	88	88	1
0	2	88	1	2	2	88	88	1

0	2	88	1	2	1	1	1	2
0	88	88	1	2	2	88	88	1
0	2	88	1	2	2	88	88	2
0	2	88	1	1	2	88	88	1
0	2	88	1	2	1	1	1	2
0	2	88	1	2	1	1	2	2
0	1	PE e nefrol	1	2	2	88	88	2
0	1	aborto	1	2	1	1	1	2
0	2	88	1	2	1	1	1	2
0	1	Prematuric	1	2	1	2	1	2
0	2	88	1	2	1	1	1	2
0	88	88	1	2	2	88	88	1
0	2	88	1	2	2	88	88	2
0	2	88	1	2	1	2	1	2
0	2	88	1	2	2	88	88	2
0	1	HPP E INFE	1	2	2	88	88	2
0	1	PE	1	2	2	88	88	2
0	2	88	1	2	2	88	88	2
0	1	HPP	1	2	2	88	88	2
0	2	88	1	2	2	88	88	2
0	2	88	1	2	2	88	88	1
0	2	88	1	2	1	1	2	2
0	88	88	1	2	1	1	1	2
0	88	88	1	1	1	3	1	1
0	1	PE	1	2	1	1	1	2
0	88	88	1	2	1	1	2	2
0	1	CURETAGE	1	2	2	88	88	1
0	2	88	1	2	2	88	88	2
0	2	88	1	2	1	1	1	2
0	88	88	1	2	1	1	1	2
0	2	88	1	1	2	88	88	1
0	88	88	1	2	2	88	88	1
0	2	88	1	2	2	88	88	2
0	2	88	1	2	2	88	88	2
0	88	88	1	2	1	2	1	1
0	88	88	1	2	2	88	88	1
0	2	88	1	2	2	88	88	2
0	88	88	1	2	2	88	88	2
0	88	88	1	2	1	2	1	2
0	2	88	1	2	1	2	1	2
0	88	88	1	2	1	2	1	2
0	2	88	1	2	1	2	1	2
0	88	88	1	2	2	88	88	1
0	2	88	1	2	1	1	2	2
0	88	88	1	2	2	88	88	1
0	2	88	1	2	2	88	88	2
0	2	88	1	2	2	88	88	2
0	1	PE E PIELOI	1	2	2	88	88	2
0	2	88	1	2	2	88	88	2
0	2	88	1	2	2	88	88	2

0	88	88	1	2	1	1	1	2
0	2	88	1	2	1	3	1	2
0	2	88	1	2	2	88	88	2
0	88	88	1	1	2	88	88	1
0	88	88	1	2	2	88	88	2
1	1	Feto mortc	1	2	2	88	88	2
0	88	88	1	2	1	1	1	2
0	2	88	1	2	2	88	88	2
0	88	88	1	2	2	88	88	2
0	2	88	1	2	1	1	1	2
0	2	88	1	2	1	2	1	2
0	2	88	1	2	1	1	1	2
0	1	Sífilis	1	2	2	88	88	2
0	2	88	1	2	1	2	1	2
0	1	Aborto esp	1	2	1	2	1	2
0	1	Prematuric	1	2	2	88	88	1
0	1	aborto	1	2	2	88	88	1
0	88	88	1	2	2	88	88	1
0	2	88	1	2	1	1	1	2
0	88	88	1	2	2	88	88	1
0	88	88	1	2	1	1	2	2
0	88	88	1	2	1	1	1	2
1	1	Ectópica	1	2	2	88	88	2
0	1	Rupreme	1	2	1	3	1	2
0	88	88	1	2	2	88	88	1
0	88	88	1	2	2	88	88	2
0	88	88	1	2	1	1	1	2
0	88	88	1	2	1	2	1	2
0	2	88	1	2	1	1	1	2
0	88	88	1	2	2	88	88	1
0	2	88	1	2	2	88	88	2
0	88	88	1	2	1	1	1	2
0	1	HAS	1	2	1	1	1	2
0	88	88	1	2	1	2	1	2
0	1	Apendicite	1	2	2	88	88	2
0	2	88	1	2	2	88	88	2
0	2	88	1	2	2	88	88	2
0	1	CURETAGE	1	2	1	1	1	2
0	2	88	1	2	2	88	88	1
0	88	88	1	2	2	88	88	1
0	88	88	1	1	1	4	1	1
0	88	88	1	2	2	88	88	1
0	2	88	1	2	2	88	88	1
0	1	HAS	2	88	88	88	88	88
0	1	PE e AVC H	2	88	88	88	88	88
0	88	88	2	88	88	88	88	88
0	2	88	2	88	88	88	88	88
0	2	88	2	88	88	88	88	88
0	1	PE	2	88	88	88	88	88
0	2	88	2	88	88	88	88	88

0	1	FETO MOR	2	88	88	88	88	88
0	88	88	2	88	88	88	88	88
0	88	88	2	88	88	88	88	88
0	88	88	2	88	88	88	88	88
0	1	PE	2	88	88	88	88	88
0	88	88	2	88	88	88	88	88
0	2	88	2	88	88	88	88	88
0	1	ACRETISMO	2	88	88	88	88	88
0	88	88	2	88	88	88	88	88
0	88	88	2	88	88	88	88	88
1	1	PE, RUPREI	2	88	88	88	88	88
0	1	GESTAÇÃO	2	88	88	88	88	88
0	88	88	2	88	88	88	88	88
0	88	88	2	88	88	88	88	88
0	1	aborto	2	88	88	88	88	88
0	88	88	2	88	88	88	88	88
0	2	88	2	88	88	88	88	88
0	88	88	2	88	88	88	88	88
0	88	88	2	88	88	88	88	88
0	88	88	2	88	88	88	88	88
0	1	DPP E HPP	2	88	88	88	88	88
0	88	88	2	88	88	88	88	88
0	2	88	2	88	88	88	88	88
0	2	88	2	88	88	88	88	88
0	88	88	2	88	88	88	88	88
0	88	88	2	88	88	88	88	88
0	88	88	2	88	88	88	88	88
0	88	88	2	88	88	88	88	88
0	2	88	2	88	88	88	88	88
88	88	88	88	88	88	88	88	88
0	1	PE	2	88	88	88	88	88
0	88	88	2	88	88	88	88	88
0	88	88	2	88	88	88	88	88
0	2	88	2	88	88	88	88	88
0	2	88	2	88	88	88	88	88
0	2	88	2	88	88	88	88	88
0	88	88	2	88	88	88	88	88
0	1	ABORTO RI	2	88	88	88	88	88
0	88	88	2	88	88	88	88	88
0	88	88	2	88	88	88	88	88
0	2	88	2	88	88	88	88	88
0	1	PE	2	88	88	88	88	88
0	88	88	2	88	88	88	88	88
0	2	88	2	88	88	88	88	88
0	88	88	2	88	88	88	88	88
0	88	88	2	88	88	88	88	88
0	2	88	2	88	88	88	88	88
88	88	88	88	88	88	88	88	88
0	88	88	2	88	88	88	88	88
88	88	88	88	88	88	88	88	88

0	2	88	2	88	88	88	88	88
0	1	DMG	2	88	88	88	88	88
0	2	88	2	88	88	88	88	88
0	2	88	2	88	88	88	88	88
0	88	88	2	88	88	88	88	88
0	1	Feto mortc	2	88	88	88	88	88
88	88	88	88	88	88	88	88	88
0	88	88	2	88	88	88	88	88
0	88	88	2	88	88	88	88	88
0	2	88	2	88	88	88	88	88
88	88	88	88	88	88	88	88	88
0	88	88	2	88	88	88	88	88
0	2	88	2	88	88	88	88	88
0	2	88	2	88	88	88	88	88
0	88	88	2	88	88	88	88	88
0	2	88	2	88	88	88	88	88
0	88	88	2	88	88	88	88	88
0	88	88	2	88	88	88	88	88
0	1	Feto mortc	2	88	88	88	88	88
0	1	PE, DPP, TC	2	88	88	88	88	88
0	2	88	2	88	88	88	88	88
0	1	PE	2	88	88	88	88	88
1	2	88	2	88	88	88	88	88
0	2	88	2	88	88	88	88	88
1	2	88	2	88	88	88	88	88
0	1	DMG, HAS	2	88	88	88	88	88
0	2	88	2	88	88	88	88	88
0	2	88	2	88	88	88	88	88
0	2	88	2	88	88	88	88	88
0	88	88	2	88	88	88	88	88
0	2	88	2	88	88	88	88	88
0	88	88	2	88	88	88	88	88
0	1	HAS, TPP. (	2	88	88	88	88	88

OCITOIVTP	OCITOIVPP	OCITOIMPI	PRESOCIIM	HEMOGLOI	HEMOGLOI	HEMATOPI	HEMATOPI	FCPP
2	2	1	1	66	66	66	66	86
2	1	1	1	66	66	66	66	73
1	1	2	1	66	66	66	66	95
1	1	1	1	66	66	66	66	100
2	1	1	1	66	66	66	66	132
1	1	1	1	66	66	66	66	93
2	2	1	1	66	66	66	66	73
1	1	1	1	66	66	66	66	92
2	2	1	1	66	66	66	66	98
1	1	1	1	66	66	66	66	84
1	1	1	1	66	66	66	66	94
2	2	1	1	66	66	66	66	98
1	1	1	1	8,9	1	66	66	101
2	2	1	1	12,9	2	36,1	2	76
2	1	1	1	66	66	66	66	64
1	1	2	1	66	66	66	66	114
1	1	1	1	11,3	1	33	1	63
2	2	1	1	66	66	66	66	107
1	1	1	1	66	66	66	66	106
1	1	1	1	7,3	1	22,1	1	90
1	1	1	1	10	1	29,5	1	84
2	2	1	1	66	66	66	66	96
1	1	2	2	66	66	66	66	116
2	2	1	1	66	66	66	66	81
1	1	1	1	66	66	66	66	99
1	1	1	1	8,9	1	66	66	78
2	1	1	1	66	66	66	66	95
2	2	1	1	66	66	66	66	59
2	1	1	1	66	66	66	66	72
1	1	1	1	66	66	66	66	97
2	1	2	1	66	66	66	66	61
2	1	1	1	13,1	2	38,7	2	92
1	1	2	1	66	66	66	66	68
1	1	1	1	66	66	66	66	72
1	1	1	1	66	66	66	66	81
2	1	1	1	66	66	66	66	85
1	1	1	1	66	66	66	66	97
1	1	1	1	66	66	66	66	111
2	1	1	2	66	66	66	66	71
2	1	1	1	66	66	66	66	86
1	1	1	1	8,3	1	24,7	1	118
1	2	1	1	66	66	66	66	99
2	2	1	1	12,6	2	38,4	2	75
1	1	1	1	66	66	66	66	108
1	1	1	1	66	66	66	66	113
1	1	1	1	66	66	66	66	80
1	1	1	1	66	66	66	66	78
2	2	1	1	66	66	66	66	77
1	1	1	1	66	66	66	66	89

1	1	1	1	66	66	66	66	110
1	1	1	1	66	66	66	66	81
2	2	1	2	66	66	66	66	70
1	1	1	1	8,8	1	25,9	1	103
1	1	1	1	66	66	66	66	77
1	1	1	1	66	66	66	66	92
1	2	1	1	66	66	66	66	70
1	1	1	1	66	66	66	66	94
2	2	1	1	9,9	1	32,2	1	81
2	2	1	1	7,8	1	24	1	94
1	1	1	1	6,5	1	20,4	1	116
1	1	1	1	66	66	66	66	77
2	1	1	1	7,7	1	21,5	1	92
2	1	1	1	66	66	66	66	92
1	1	1	1	66	66	66	66	111
1	1	1	1	66	66	66	66	87
1	1	1	1	9	1	26,3	1	118
2	2	1	1	66	66	66	66	75
1	1	1	1	66	66	66	66	84
2	1	1	1	66	66	66	66	86
1	1	1	1	66	66	66	66	82
2	2	1	1	66	66	66	66	60
2	2	1	1	10,4	1	31,3	1	78
2	2	1	1	9,9	1	32,2	1	81
1	1	1	1	66	66	66	66	84
1	2	2	1	66	66	66	66	87
1	1	1	1	66	66	66	66	84
2	2	1	1	66	66	66	66	96
2	2	1	1	66	66	66	66	67
1	1	1	1	66	66	66	66	81
2	2	2	1	66	66	66	66	92
2	2	1	1	66	66	66	66	79
2	2	1	1	66	66	66	66	95
1	1	1	1	66	66	66	66	82
2	2	1	1	66	66	66	66	96
2	1	1	1	66	66	66	66	84
2	1	1	1	66	66	66	66	88
2	1	1	1	11,9	2	34,4	1	64
1	1	1	1	66	66	66	66	70
1	1	2	1	66	66	66	66	74
1	1	1	1	66	66	66	66	97
2	1	2	2	66	66	66	66	72
1	1	2	1	66	66	66	66	107
1	1	1	1	66	66	66	66	106
1	1	1	1	66	66	66	66	126
2	2	1	1	66	66	66	66	77
1	1	1	1	66	66	66	66	88
1	1	1	1	66	66	66	66	108
1	1	1	1	66	66	66	66	70
2	1	1	1	66	66	66	66	76

2	2	1	1	66	66	66	66	78
1	1	1	1	66	66	66	66	94
2	2	1	1	66	66	66	66	74
1	1	1	1	8,5	1	66	66	70
1	1	1	1	9,5	1	28,3	1	84
2	1	2	1	11,9	2	34,1	1	76
1	1	1	1	11,5	2	66	66	64
1	2	2	1	66	66	66	66	124
2	1	1	1	66	66	66	66	60
1	1	1	1	66	66	66	66	123
2	1	1	1	66	66	66	66	102
1	2	1	1	66	66	66	66	86
2	1	2	1	11,9	2	35,2	1	55
1	1	1	1	66	66	66	66	71
1	1	2	2	11,1	1	32	1	75
1	1	1	1	66	66	66	66	109
1	1	1	1	66	66	66	66	66
2	1	1	1	66	66	66	66	78
2	2	1	1	66	66	66	66	90
2	2	1	1	66	66	66	66	67
1	1	1	1	66	66	66	66	85
1	1	1	1	66	66	66	66	58
1	1	1	2	7,4	1	22,8	1	90
1	1	1	1	8,9	1	26,8	1	79
1	1	1	1	66	66	66	66	78
2	1	2	2	66	66	66	66	81
1	1	1	1	66	66	66	66	89
2	1	1	1	66	66	66	66	70
2	1	1	1	66	66	66	66	46
2	1	1	1	66	66	66	66	85
1	1	1	1	8,2	1	25	1	146
1	1	1	1	66	66	66	66	114
1	1	1	1	66	66	66	66	92
2	1	1	1	66	66	66	66	58
1	1	1	1	66	66	66	66	95
1	2	1	2	66	66	66	66	90
2	2	1	1	66	66	66	66	62
1	1	1	1	66	66	66	66	118
2	1	1	1	66	66	66	66	82
1	1	1	1	66	66	66	66	71
2	2	1	1	66	66	66	66	62
2	1	1	2	66	66	66	66	107
1	1	1	1	66	66	66	66	86
1	1	1	1	66	66	66	66	72
2	1	1	2	66	66	66	66	89
2	1	1	1	66	66	66	66	64
1	1	1	1	66	66	66	66	89
2	1	1	1	66	66	66	66	70
2	2	2	1	66	66	66	66	99
1	1	2	1	66	66	66	66	83

2	1	1	1	66	66	66	66	79
2	1	1	1	66	66	66	66	86
1	1	1	1	66	66	66	66	80
1	1	2	1	9,5	1	66	66	99
1	1	1	1	66	66	66	66	80
1	2	1	1	10,5	1	31,4	1	98
1	1	1	1	66	66	66	66	101
2	1	1	1	66	66	66	66	71
1	1	1	1	12,4	2	37,1	2	62
1	1	1	1	66	66	66	66	85
1	1	1	#NULL!	66	66	66	66	91
2	1	1	1	66	66	66	66	71
2	2	1	1	66	66	66	66	99
1	1	2	1	66	66	66	66	71
2	1	1	1	66	66	66	66	86
1	1	2	1	66	66	66	66	69
1	2	1	1	66	66	66	66	98
1	1	1	1	8	1	23,9	1	126
1	1	1	1	66	66	66	66	91
2	1	1	1	66	66	66	66	104
1	1	2	2	66	66	66	66	73
1	1	1	1	66	66	66	66	72
1	1	2	2	66	66	66	66	66
2	1	1	1	66	66	66	66	111
1	1	2	1	66	66	66	66	72
1	1	1	1	66	66	66	66	78
2	1	1	1	66	66	66	66	102
1	1	1	1	66	66	66	66	94
2	2	1	1	66	66	66	66	84
1	1	2	1	66	66	66	66	68
1	1	1	1	66	66	66	66	84
1	1	1	1	66	66	66	66	103
1	1	2	1	66	66	66	66	99
2	1	1	1	13,2	2	38,5	2	88
1	1	1	1	66	66	66	66	82
1	1	1	2	66	66	66	66	52
2	1	2	1	10,5	1	31,7	1	80
2	1	1	1	66	66	66	66	70
1	1	2	1	10,3	1	29,2	1	62
1	1	2	1	66	66	66	66	94
1	1	1	1	66	66	66	66	87
1	1	1	1	8,8	1	23,6	1	95
2	1	1	1	66	66	66	66	76
2	1	2	2	66	66	66	66	92
2	1	2	2	66	66	66	66	62
1	1	2	2	66	66	66	66	80
2	1	2	2	11,9	2	35,6	2	83
2	1	2	2	66	66	66	66	61
2	1	2	2	66	66	66	66	66
2	1	2	2	66	66	66	66	74

2	1	2	2	66	66	66	66	68
1	1	2	2	66	66	66	66	62
1	1	2	2	11,5	2	36,1	2	79
2	1	2	2	66	66	66	66	67
2	1	2	2	66	66	66	66	80
1	1	2	2	66	66	66	66	91
2	1	2	2	66	66	66	66	56
2	1	2	2	66	66	66	66	67
1	1	2	2	8,5	1	25,2	1	82
1	1	2	2	8,7	1	26,3	1	70
2	1	2	2	66	66	66	66	75
2	1	2	2	11,6	2	34,9	1	63
1	1	2	2	66	66	66	66	85
1	1	2	1	66	66	66	66	89
1	1	2	2	66	66	66	66	87
1	1	2	2	66	66	66	66	72
2	1	2	2	66	66	66	66	64
1	1	2	2	66	66	66	66	70
1	1	2	2	66	66	66	66	63
1	1	2	2	66	66	66	66	88
2	1	2	2	66	66	66	66	73
1	1	2	2	9,1	1	26,6	1	78
2	1	2	2	66	66	66	66	74
2	1	2	2	9,3	1	29,4	1	68
1	1	2	2	66	66	66	66	59
2	1	2	2	66	66	66	66	89
1	1	2	1	66	66	66	66	65
2	1	2	2	66	66	66	66	67
2	1	2	2	66	66	66	66	72
88	88	88	88	88	88	88	88	888
2	1	2	2	66	66	66	66	82
1	1	2	2	66	66	66	66	54
2	1	2	2	66	66	66	66	79
2	1	2	2	66	66	66	66	98
2	1	2	2	66	66	66	66	74
1	1	2	2	6,5	1	18,5	1	64
1	1	2	2	8,4	1	24,6	2	79
1	1	2	2	66	66	66	66	81
2	1	2	2	66	66	66	66	82
1	1	2	2	66	66	66	66	96
1	1	2	2	66	66	66	66	72
2	1	2	2	11	1	31,9	1	99
1	1	2	2	66	66	66	66	55
1	1	2	2	9,5	1	27,5	1	81
1	1	2	2	14,2	2	41,4	2	96
1	1	2	2	66	66	66	66	82
2	1	2	2	10,4	1	33,1	1	60
88	88	88	88	88	88	88	88	888
2	1	2	2	66	66	66	66	72
88	88	88	88	88	88	88	88	888

1	1	2	2	66	66	66	66	101
2	1	2	2	11,7	2	35,2	1	97
2	1	2	2	7,7	1	66	66	85
2	1	2	2	66	66	66	66	68
1	1	2	2	9,5	1	28,8	1	71
2	1	2	2	66	66	66	66	73
88	88	88	88	88	88	88	88	888
1	1	2	2	66	66	66	66	69
1	1	2	2	66	66	66	66	86
2	1	2	1	11	1	33,5	1	84
88	88	88	88	88	88	88	88	888
1	1	2	2	66	66	66	66	104
2	1	2	2	7,5	1	22,9	2	91
2	1	2	2	66	66	66	66	62
1	1	2	2	11,8	2	36,2	2	74
1	1	2	2	10	1	30,3	1	116
1	1	2	2	66	66	66	66	80
1	1	2	2	66	66	66	66	75
2	1	2	2	66	66	66	66	79
1	1	2	2	8,2	1	23,2	1	65
1	1	2	2	66	66	66	66	73
1	1	2	2	66	66	66	66	122
2	1	2	2	66	66	66	66	56
2	1	2	2	66	66	66	66	82
1	1	2	2	66	66	66	66	73
2	1	2	2	66	66	66	66	73
1	1	2	2	66	66	66	66	72
2	1	2	2	66	66	66	66	79
2	1	2	2	66	66	66	66	64
1	1	2	2	66	66	66	66	79
2	1	2	2	66	66	66	66	80
1	1	2	2	66	66	66	66	73
2	1	2	2	66	66	66	66	62

PASPP	ICHO	ICHOCAT	CLAMP	AMPH	PESORN	ADEPESORI	HPPHP	HPPDISTUT
138	0,62	1	1	1	3200	2	2	2
108	0,68	1	2	1	3010	2	88	2
91	1,04	2	1	2	2365	1	2	2
140	0,71	1	2	2	3780	2	2	2
144	0,91	2	2	2	3255	2	2	2
123	0,75	1	2	1	3910	2	2	2
149	0,48	1	2	1	3235	2	2	2
105	0,87	1	1	1	3470	2	2	2
93	1,05	2	2	1	2955	2	2	2
119	0,7	1	2	2	3260	2	1	1
140	0,67	1	2	1	3630	2	2	2
93	1,05	2	2	1	2955	2	2	2
106	0,95	2	2	2	3430	2	2	2
97	0,78	1	1	1	2830	2	2	2
124	0,51	1	2	2	3830	2	2	2
104	1,09	2	2	2	2760	2	88	2
86	0,73	1	1	2	2930	2	2	2
94	1,13	2	2	1	3660	2	2	2
92	1,15	2	2	2	2865	2	2	2
114	0,78	1	2	2	3365	2	88	2
103	0,81	1	2	1	2645	1	2	2
122	0,78	1	2	2	3530	2	88	2
106	1,09	2	2	1	2505	1	88	2
116	0,69	1	2	1	3390	2	2	2
126	0,78	1	2	2	2605	1	88	2
116	0,67	1	2	2	4155	3	1	1
140	0,67	1	2	1	3045	2	2	2
106	0,55	1	2	1	3715	2	2	2
111	0,64	1	2	1	3410	2	2	2
102	0,88	1	2	1	3065	2	88	2
112	0,54	1	2	1	4052	2	2	2
123	0,74	1	2	2	2785	1	2	2
99	0,68	1	2	2	3240	2	88	2
112	0,64	1	2	2	3260	2	2	2
106	0,76	1	2	1	3355	2	88	2
113	0,75	1	2	1	2670	1	2	2
160	0,6	1	1	1	2640	2	2	2
148	0,75	1	1	2	3010	2	2	2
94	0,75	1	2	1	3855	2	2	2
122	0,7	1	2	2	3230	2	88	2
96	1,22	2	2	2	3590	2	88	2
136	0,72	1	1	1	3855	2	2	2
99	0,75	1	2	1	3630	2	2	2
105	1,02	2	1	2	3125	2	2	2
128	0,88	1	2	1	2755	2	88	2
126	0,63	1	1	1	3000	2	2	2
112	0,69	1	2	2	4068	2	2	2
102	0,75	1	2	1	3410	2	2	2
103	0,86	1	2	1	3635	2	2	2

80	1,37	2	1	2	2225	1	88	2
146	0,55	1	2	1	2705	2	2	2
93	0,75	1	2	1	3425	2	2	2
144	0,71	1	2	1	3130	2	88	2
116	0,66	1	2	1	3945	2	88	2
128	0,71	1	1	2	3300	2	2	2
124	0,56	1	2	2	2915	2	88	2
126	0,74	1	1	2	3455	2	88	2
147	0,55	1	2	1	3410	2	2	2
163	0,57	1	2	1	2575	1	2	2
112	1,04	2	2	1	3285	2	88	2
141	0,54	1	1	1	4190	3	2	1
100	0,92	2	2	1	3040	2	88	2
88	1,04	2	1	1	2982	2	88	2
163	0,68	1	2	1	3680	2	88	2
111	0,78	1	2	1	3075	2	2	2
104	1,13	2	2	2	2950	2	88	2
95	0,78	1	1	2	2892	1	2	2
115	0,73	1	1	1	2720	1	88	2
122	0,7	1	1	1	3470	2	2	2
122	0,72	1	2	1	3440	2	2	2
101	0,59	1	1	2	3565	2	2	2
110	0,71	1	2	2	3895	2	1	2
147	0,55	1	2	1	3410	2	2	2
120	0,7	1	2	1	2260	1	2	2
130	0,66	1	2	1	3590	2	88	2
160	0,52	1	1	1	3240	2	2	2
112	0,85	1	2	1	2715	2	2	2
129	0,51	1	2	1	2625	1	2	2
88	0,92	2	1	2	3022	2	88	2
100	0,92	2	2	2	3255	2	2	2
93	0,84	1	2	1	3730	2	2	2
102	0,87	1	1	1	2790	1	2	2
100	0,82	1	2	1	3122	2	2	2
108	0,88	1	2	1	3225	2	88	2
134	0,62	1	1	1	4285	3	2	1
121	0,72	1	2	2	3030	2	88	2
107	0,59	1	1	2	3100	2	2	2
128	0,55	1	2	1	2890	2	2	2
114	0,56	1	2	1	2792	2	2	2
125	0,78	1	1	1	3505	2	88	2
107	0,67	1	1	1	3460	2	2	2
106	1,01	2	2	2	3415	2	88	2
105	1,01	2	2	1	3480	2	88	2
127	0,99	2	2	2	3190	2	2	2
118	0,65	1	1	1	3804	2	2	2
144	0,61	1	2	1	3560	2	2	2
127	0,85	1	1	2	3200	2	88	2
100	0,7	1	2	1	3065	2	88	2
134	0,57	1	2	1	3625	2	2	2

124	0,62	1	1	1	3515	2	2	2
97	0,96	2	1	2	3330	2	88	2
108	0,69	1	2	2	3150	2	2	2
83	0,84	1	2	1	3260	2	2	2
98	0,85	1	2	1	3735	2	2	2
110	0,69	1	2	2	3285	2	2	2
132	0,42	1	2	2	2330	1	2	2
106	1,16	2	2	2	2715	2	2	2
110	0,54	1	2	1	3200	2	2	2
129	0,95	2	1	1	3710	2	2	2
142	0,72	1	1	1	3330	2	2	2
108	0,79	1	1	1	3485	2	88	2
121	0,45	1	2	1	3135	2	2	2
113	0,62	1	2	2	3195	2	2	2
129	0,58	1	1	1	3025	2	2	2
135	0,8	1	2	2	3810	2	1	2
121	0,54	1	2	2	3260	2	2	2
101	0,77	1	1	1	3160	2	2	2
139	0,65	1	1	1	3660	2	1	2
134	0,5	1	2	1	2580	1	2	2
111	0,76	1	2	1	2845	2	2	2
96	0,67	1	1	2	2595	1	2	2
117	0,76	1	1	2	3790	2	88	2
117	0,67	1	1	1	3475	2	88	2
119	0,65	1	2	2	3525	2	2	2
118	0,69	1	2	1	2850	2	88	2
134	0,66	1	1	2	3467	2	2	2
143	0,48	1	2	66	3425	2	2	2
108	0,42	1	2	2	3275	2	2	2
99	0,85	1	2	1	4100	3	88	1
98	1,48	2	1	1	4150	1	2	1
117	0,97	2	2	2	2935	2	88	2
118	0,77	1	2	1	3400	2	2	2
146	0,39	1	2	1	2985	2	2	2
114	0,83	1	2	1	3425	2	88	2
102	0,88	1	1	2	2870	2	88	2
148	0,41	1	2	1	2840	1	2	2
125	0,94	2	2	1	2955	2	88	2
138	0,59	1	2	2	2480	1	88	2
100	0,71	1	2	2	3395	2	2	2
121	0,51	1	2	2	2685	1	88	2
137	0,78	1	1	1	3390	2	2	2
111	0,77	1	2	1	32335	2	88	2
119	0,6	1	2	1	3566	3	2	1
118	0,75	1	2	1	3155	2	88	2
167	0,38	1	2	2	3615	2	2	2
117	0,76	1	2	1	3190	2	2	2
110	0,63	1	1	2	3204	2	2	2
129	0,76	1	1	2	3040	2	2	2
133	0,67	1	2	1	3000	2	2	2

119	0,66	1	2	1	2795	1	88	2
97	0,89	1	1	2	2940	2	2	2
105	0,76	1	2	1	3295	2	2	2
100	0,99	2	1	1	3655	2	88	2
123	0,65	1	2	2	2910	2	88	2
93	1,05	2	2	1	3715	2	1	2
166	0,6	1	1	2	2910	2	88	2
127	0,55	1	2	1	3010	2	2	2
148	0,41	1	2	1	2585	2	88	2
120	0,7	1	2	1	4120	3	2	1
107	0,85	1	2	1	3325	2	2	2
125	0,56	1	2	1	2790	1	2	2
119	0,83	1	1	1	4180	3	2	1
110	0,64	1	2	1	3040	2	2	2
131	0,65	1	2	1	3725	2	1	2
98	0,7	1	2	1	3075	2	2	2
91	1,07	2	1	2	3310	2	2	2
107	1,17	2	1	1	3520	2	88	2
115	0,79	1	2	1	3160	2	2	2
142	0,73	1	2	2	2915	2	88	2
117	0,62	1	2	2	3235	2	88	2
106	0,67	1	2	1	3025	2	88	2
93	0,7	1	2	1	3825	2	2	2
123	0,9	2	2	1	3410	2	2	2
141	0,51	1	1	1	2890	2	88	2
107	0,72	1	2	1	3150	2	88	2
123	0,82	1	2	1	3580	2	88	2
144	0,65	1	2	1	3235	2	88	2
97	0,86	1	2	2	3300	2	2	2
90	0,75	1	2	1	2700	1	88	2
162	0,51	1	1	2	2700	1	2	2
135	0,76	1	1	1	3300	2	88	2
143	0,69	1	2	1	3730	2	2	2
116	0,75	1	2	2	2325	2	88	2
116	0,7	1	2	1	3395	2	2	2
90	0,57	1	2	1	3310	2	2	2
77	1,03	2	2	1	4485	3	2	1
103	0,67	1	2	1	3180	2	2	2
91	0,68	1	1	1	2670	1	2	2
120	0,78	1	2	1	3805	2	88	2
117	0,74	1	2	2	4570	3	88	1
99	0,95	2	2	2	3400	2	88	2
104	0,73	1	2	2	3750	2	2	2
132	0,69	1	2	1	3765	2	2	2
105	0,58	1	2	1	2640	2	2	2
127	0,62	1	2	1	3300	2	88	2
94	0,88	1	2	1	2800	2	2	2
121	0,5	1	2	1	2970	2	2	2
95	0,69	1	2	2	3635	2	2	2
112	0,59	1	2	2	3370	2	2	2

107	0,63	1	1	1	3254	2	2	2
98	0,63	1	1	2	2895	1	88	2
136	0,58	1	2	1	4065	3	88	1
119	0,56	1	1	1	2885	2	88	2
120	0,66	1	1	2	3565	2	2	2
105	0,86	1	1	2	3645	2	88	2
132	0,41	1	1	2	2190	1	2	2
98	0,68	1	1	2	3660	2	1	2
100	0,82	1	2	2	3635	2	88	2
108	0,64	1	1	2	3040	2	88	2
118	0,63	1	1	2	2545	1	2	2
11	0,56	1	1	2	3075	2	2	2
96	0,88	1	1	2	3500	2	88	2
119	0,74	1	66	2	3515	2	88	2
97	0,89	1	2	2	3320	2	2	2
113	0,63	1	1	2	2600	2	88	2
133	0,48	1	1	2	2047	1	2	2
108	0,64	1	1	1	3770	2	88	2
108	0,58	1	2	1	4165	3	88	1
105	0,84	1	1	2	3215	2	88	2
116	0,62	1	1	1	3295	2	2	2
140	0,55	1	2	2	2290	1	88	2
116	0,63	1	1	2	3109	2	2	2
162	0,41	1	2	1	3305	2	2	2
104	0,56	1	1	2	4000	2	88	2
107	0,83	1	2	2	3445	2	88	2
96	0,67	1	1	2	3125	2	88	2
101	0,66	1	2	1	2250	1	88	2
103	0,69	1	1	2	3426	2	2	1
888	88	88	1	2	3050	2	88	88
110	0,75	1	1	2	3025	2	2	2
121	0,45	1	1	2	3855	2	88	2
117	0,68	1	1	1	3280	2	88	2
102	0,96	2	1	2	3220	2	2	2
93	0,79	1	2	2	3635	2	2	2
122	0,52	1	1	2	3810	2	2	2
117	0,67	1	1	2	4290	3	88	1
121	0,66	1	1	2	3990	2	2	2
113	0,72	1	2	2	2800	1	88	2
127	0,76	1	2	2	3780	2	88	2
92	0,78	1	1	2	3452	2	2	2
101	0,98	2	2	2	3390	2	2	2
106	0,51	1	1	2	3880	2	88	2
98	0,82	1	1	1	3675	2	2	2
129	0,74	1	2	2	3230	2	88	2
88	0,93	2	66	2	3225	2	88	2
126	0,47	1	1	2	2250	1	2	1
888	88	88	1	2	2494	1	88	88
120	0,6	1	1	2	2345	2	88	1
888	88	88	2	2	2468	2	88	88

107	0,94	2	2	1	4065	3	2	1
112	0,86	1	1	2	4340	3	2	1
124	0,68	1	2	2	3875	2	2	2
98	0,69	1	2	1	3934	2	2	2
102	0,69	1	1	2	2930	2	88	2
105	0,69	1	1	1	2260	1	2	1
888	88	88	2	2	2374	1	88	88
94	0,73	1	2	1	3385	2	88	2
107	0,8	1	2	2	3350	2	88	2
112	0,75	1	2	2	2345	2	2	1
888	88	88	2	2	2560	2	88	88
124	0,83	1	1	2	3610	2	88	2
97	0,93	2	1	2	2468	1	2	2
105	0,59	1	1	2	2585	2	2	2
126	0,58	1	1	2	3185	2	88	2
136	0,85	1	1	2	3455	2	2	2
128	0,62	1	1	2	3420	2	88	2
128	0,58	1	2	1	3970	2	88	2
106	0,74	1	2	2	4250	3	2	1
88	0,73	1	2	1	3330	2	2	2
118	0,62	1	1	2	3810	3	2	2
149	0,82	1	1	2	3680	2	2	2
104	0,53	1	1	1	3000	2	2	2
100	0,82	1	1	2	3250	2	2	1
104	0,7	1	2	2	3850	2	2	2
100	0,73	1	2	2	4605	3	2	2
129	0,55	1	2	1	4515	3	2	1
105	0,75	1	2	2	3480	2	2	2
94	0,68	1	1	2	4040	3	2	1
110	0,71	1	2	2	4135	3	88	1
130	0,61	1	1	2	2995	2	2	2
122	0,59	1	1	1	2910	2	88	2
118	0,52	1	1	2	1960	1	2	2

HPPDISTCC	HPPANTICC	HPPCESPRE	HPPPLACPA	HPPMULTG	HPPELEVNI	HPPANEMI	HPPPRIMFI	HPPPARTPI
2	2	88	2	2	2	66	88	2
2	2	88	2	2	2	66	88	2
2	2	88	2	2	1	2	2	2
2	2	88	2	2	2	1	88	2
2	2	88	2	2	2	2	88	2
2	2	88	2	2	2	2	88	2
2	2	88	2	2	2	2	88	2
2	2	88	2	2	2	2	88	2
2	2	88	2	2	2	2	88	2
2	2	88	2	2	2	66	88	2
2	2	88	2	2	2	66	88	2
2	2	88	2	2	2	2	88	2
2	2	88	2	2	2	2	88	2
2	2	88	2	2	2	66	88	2
2	2	88	2	2	2	66	88	2
2	2	88	2	2	2	2	88	2
2	2	88	2	2	2	66	88	2
2	2	88	2	2	2	66	88	2
2	2	88	2	2	2	1	88	2
2	2	88	2	2	2	2	88	2
2	2	88	2	2	2	66	88	2
2	2	88	2	2	2	2	88	2
2	2	88	2	2	2	2	88	2
2	2	88	2	2	2	2	88	2
2	2	88	2	2	2	2	88	2
2	2	88	2	1	2	2	88	2
2	2	88	2	2	2	66	88	2
2	2	2	2	2	2	1	88	2
2	2	88	2	2	2	66	88	2
2	2	88	2	2	2	66	88	2
2	2	88	2	2	2	66	88	2
2	2	88	2	2	1	66	88	2
2	2	88	2	2	2	2	88	2
2	2	2	2	2	2	2	88	2
2	2	88	2	2	2	66	88	2
2	2	88	2	2	2	66	88	2
2	2	88	2	2	1	2	88	2
2	2	2	2	2	2	2	88	2
2	2	88	2	2	2	66	88	2
2	2	88	2	2	2	66	88	2
2	2	88	2	2	2	66	88	2
2	2	88	2	2	2	2	88	2
2	2	2	2	2	2	66	88	2
2	2	88	2	2	2	2	88	2
2	2	88	2	2	2	2	88	2
2	2	88	2	2	1	2	88	2
2	2	88	2	2	2	66	88	2
2	2	88	2	2	2	66	88	2
2	2	88	2	2	2	2	88	2

2	2	88	2	2	2	2	88	2
2	2	2	2	2	1	2	88	2
2	2	88	2	2	2	66	88	2
2	2	88	2	2	2	2	88	2
2	2	88	2	2	2	1	88	2
2	2	88	2	2	2	2	88	2
2	2	88	2	2	2	1	88	2
2	2	88	2	2	2	66	88	2
2	2	88	2	2	1	66	88	2
2	2	88	2	2	1	2	88	2
2	2	88	2	2	2	66	88	2
2	2	88	2	1	2	1	88	2
2	2	88	2	2	2	66	88	2
2	2	88	2	2	2	66	88	2
2	2	88	2	2	2	66	88	2
2	2	88	2	2	2	2	88	2
2	2	88	2	2	2	2	88	2
2	2	88	2	2	2	2	88	2
2	2	88	2	2	2	2	88	2
2	2	88	2	2	2	66	88	2
2	2	88	2	2	2	2	88	2
2	2	88	2	2	2	66	88	2
2	2	88	2	2	2	66	88	2
2	2	88	2	2	1	66	88	2
2	2	88	2	2	2	2	88	2
2	2	88	2	2	2	2	88	2
2	2	88	2	2	1	2	88	2
2	2	88	2	1	2	2	88	2
2	2	2	2	2	2	2	88	2
2	2	88	2	2	1	66	88	2
2	2	88	2	2	2	2	88	2
2	2	88	2	2	2	66	88	2
2	2	88	2	2	2	1	88	2
2	2	88	2	2	2	66	88	2
2	2	88	2	2	2	2	88	2
2	2	2	2	2	2	2	88	2
2	2	88	2	2	2	2	88	2
2	2	88	2	2	2	66	88	2
2	2	88	2	2	2	66	88	2
2	2	88	2	2	2	2	88	2
2	2	88	2	2	2	66	88	2
2	2	88	2	2	2	2	88	2
2	2	88	2	2	2	2	88	2
2	2	88	2	2	2	66	88	2
2	2	88	2	2	1	2	88	2
2	2	88	2	2	2	1	88	2
2	2	88	2	2	2	66	88	2
2	2	88	2	2	2	66	88	2
2	2	88	2	2	2	2	88	2
2	2	88	2	2	2	66	88	2

2	2	88	2	2	2	66	88	2
2	2	88	2	2	2	2	88	2
2	2	88	2	2	2	66	88	2
2	2	88	2	2	2	2	88	2
2	2	88	2	2	2	1	88	2
2	2	88	2	2	2	66	88	2
2	2	88	2	1	1	2	88	2
2	2	88	2	2	1	1	88	2
2	2	88	2	2	2	2	88	2
2	2	88	2	2	2	1	88	2
2	2	88	2	2	2	2	88	2
2	2	88	2	2	2	66	88	2
2	2	88	2	2	2	66	88	2
2	2	88	2	2	2	2	88	2
2	2	88	2	2	2	66	88	2
2	2	88	2	2	2	2	88	2
2	2	88	2	2	2	2	88	2
2	2	88	2	2	2	66	88	2
2	2	88	2	2	2	2	88	2
2	2	88	2	2	2	66	88	2
2	2	2	2	2	2	2	88	2
2	2	88	2	2	2	66	88	2
2	2	88	2	2	2	66	88	2
2	2	88	2	2	2	66	88	2
2	2	88	2	2	1	1	88	2
2	2	88	2	2	2	66	88	2
2	2	88	2	2	1	2	88	2
2	2	88	2	2	2	66	88	2
2	2	2	2	2	2	2	88	2
2	2	88	2	2	2	2	88	2
2	2	88	2	2	2	66	88	2
2	2	88	2	2	2	1	88	2
2	2	88	2	2	2	2	88	2
2	2	88	2	1	1	2	88	2
2	2	88	2	2	2	2	88	2
2	2	88	2	2	2	2	1	2
2	2	88	2	1	2	66	88	2
2	2	88	2	2	2	2	88	2
2	2	88	2	2	2	2	88	2
2	2	88	2	2	2	66	88	2
2	2	88	2	2	2	2	88	2
2	2	88	2	2	2	2	88	2
2	2	88	2	2	2	66	88	2
2	2	88	2	2	1	2	88	2
2	2	88	2	2	2	66	88	2
2	2	88	2	2	1	2	88	2
2	2	88	2	2	2	2	88	2
2	2	88	2	2	2	66	88	2
2	2	2	2	2	2	66	88	2
2	2	88	2	1	2	2	88	2

2	2	88	2	2	2	2	88	2
2	2	88	2	2	2	66	88	2
2	2	88	2	1	2	2	88	2
2	2	88	2	2	1	66	88	2
2	2	88	2	2	2	2	88	2
2	2	88	2	2	2	1	88	2
2	2	88	2	2	2	2	88	2
2	2	88	2	2	2	2	88	2
2	2	88	2	2	1	2	88	2
2	2	88	2	2	2	2	88	2
2	2	88	2	2	2	1	88	2
2	2	88	2	2	2	66	88	2
2	2	88	2	2	2	2	88	2
2	2	88	2	2	2	2	88	2
2	2	88	2	2	2	66	88	2
2	2	88	2	2	2	2	88	2
2	2	88	2	2	2	1	88	2
2	2	88	2	2	2	2	88	2
2	2	2	2	2	2	2	88	2
2	2	88	2	2	1	2	88	2
2	2	88	2	2	2	2	88	2
2	2	88	2	2	2	66	88	2
2	2	88	2	2	2	2	2	2
2	2	2	2	2	2	66	88	2
2	2	88	2	2	2	66	88	2
2	2	88	2	2	2	1	88	2
2	2	88	2	2	2	66	88	2
2	2	88	2	2	1	2	88	2
2	2	88	2	2	2	2	88	2
2	2	88	2	2	2	66	88	2
2	2	88	2	2	1	1	88	2
2	2	88	2	2	2	66	88	2
2	2	88	2	1	2	2	88	2
2	2	88	2	2	2	66	88	2
2	2	88	2	2	2	1	88	2
2	2	88	2	2	2	66	88	2
2	2	88	2	2	2	2	88	2
2	2	88	2	2	2	88	88	2
2	2	88	2	2	2	2	88	2
2	2	88	2	2	1	2	88	2
2	2	88	2	2	2	66	88	2
2	2	88	2	2	2	2	88	2
2	2	88	2	2	2	66	88	2
2	2	2	2	2	1	2	88	2
2	2	2	2	1	1	1	88	2
2	2	88	2	2	2	2	88	2
2	2	2	2	2	2	66	88	2
2	2	2	2	2	1	2	88	2
2	2	2	2	2	2	2	88	2
2	2	2	2	2	2	2	88	2

2	2	88	2	2	2	66	88	2
2	2	88	2	2	2	2	88	2
2	2	88	2	2	2	2	88	2
2	2	88	2	2	2	2	88	2
2	2	2	2	2	1	1	88	2
2	2	88	2	2	2	2	88	2
2	2	2	2	1	2	66	88	2
2	2	2	2	1	2	2	88	2
2	2	88	2	2	2	2	88	2
2	2	88	2	2	2	66	88	2
2	2	2	2	2	2	2	88	2
2	2	2	2	2	2	1	88	2
2	2	88	2	2	2	2	88	2
2	2	88	2	2	2	1	88	2
2	2	88	2	2	2	2	88	2
2	2	88	2	2	1	2	88	2
2	2	2	2	2	2	2	88	2
2	2	88	2	2	2	2	88	2
2	2	88	2	2	2	2	88	2
2	2	88	2	2	2	2	88	2
2	1	2	2	2	2	2	88	2
2	2	88	2	2	2	66	88	2
2	1	88	2	2	1	2	88	2
2	2	88	2	2	2	1	88	2
2	2	88	2	2	2	2	88	2
2	2	88	2	2	2	66	88	2
2	2	88	2	2	1	2	88	2
1	1	88	2	2	2	2	88	2
2	2	2	2	2	2	2	88	2
88	88	88	88	88	88	88	88	88
2	2	2	2	2	2	2	2	2
2	2	88	2	2	2	1	88	2
2	2	88	2	2	2	2	88	2
2	2	88	2	2	2	66	88	2
2	2	2	2	2	2	2	88	2
2	2	88	2	2	2	1	88	2
2	2	88	2	2	2	66	88	2
2	2	88	2	2	2	2	88	2
2	2	88	2	2	2	66	88	2
2	2	88	2	2	2	1	88	2
2	2	88	2	2	2	1	88	2
2	2	2	2	2	2	2	88	2
2	2	88	2	2	2	66	88	2
2	2	2	2	2	2	2	88	2
2	2	88	2	2	2	2	88	2
2	2	88	2	2	2	2	88	2
2	2	2	2	2	2	66	88	2
88	88	88	88	88	88	88	88	88
2	2	88	2	2	2	2	88	2
88	88	88	88	88	88	88	88	88

2	2	2	2	2	2	2	88	2
2	1	2	2	2	1	2	88	2
2	2	2	2	2	2	1	88	2
2	2	2	2	2	2	2	2	2
2	2	88	2	2	2	2	88	2
2	1	88	2	2	2	1	88	2
88	88	88	88	88	88	88	88	88
2	2	88	2	2	1	1	88	2
2	2	88	2	2	2	2	88	2
2	2	2	2	2	2	66	88	2
88	88	88	88	88	88	88	88	88
2	2	88	2	2	2	2	88	2
2	2	2	2	2	2	2	88	2
2	2	2	2	2	2	2	88	2
2	2	88	2	2	1	2	88	2
2	2	88	2	2	2	66	88	2
2	2	88	2	2	2	66	88	2
2	2	88	2	2	1	2	88	2
2	2	88	2	2	2	2	88	2
2	2	2	2	2	2	66	88	2
2	1	88	2	2	2	2	88	2
2	2	88	2	2	2	1	88	2
2	2	88	2	2	2	1	88	2
2	2	2	2	1	2	2	88	2
2	2	88	2	2	2	66	88	2
1	1	2	2	2	2	2	88	2
2	2	88	2	2	2	2	88	2
2	2	2	2	2	2	2	2	2
2	2	2	2	2	2	1	2	2
2	2	88	2	2	2	2	88	2
2	2	2	2	2	2	2	88	2
2	2	88	2	2	1	66	88	2
2	2	2	2	2	1	2	88	2

HPPTPTAQ	HPPTRAUN	HPPPLACPI	HPPDPP	HPPPARTIN	HPPCORIO/	HPPPPPC	HPPPARTIN	ESTRATIFIC
2	2	2	2	2	2	2	2	1
66	2	2	2	2	2	2	2	66
1	2	2	2	1	2	2	2	2
2	2	2	2	2	2	2	2	1
88	88	2	2	2	2	2	88	1
2	1	2	2	2	2	2	2	1
1	2	2	2	2	2	2	2	2
2	2	2	2	2	2	2	2	1
2	2	2	2	2	2	2	2	2
2	2	2	2	2	2	2	2	2
2	2	2	2	2	2	2	2	1
2	2	2	2	2	2	2	2	2
2	2	2	2	2	2	2	2	1
1	2	2	2	2	2	2	2	1
1	2	2	2	2	2	2	2	2
2	2	2	2	2	2	2	2	1
2	2	2	2	2	2	2	2	1
2	2	2	2	2	2	2	2	1
2	2	2	2	2	2	2	2	1
2	2	2	2	1	2	2	2	1
2	2	2	2	2	2	2	2	2
1	2	2	2	2	2	2	2	1
2	2	2	2	2	2	2	2	66
2	2	2	2	2	2	2	2	2
2	2	2	2	1	2	2	2	2
2	2	2	2	2	2	2	2	2
2	2	2	2	2	2	2	2	1
2	2	2	2	2	2	2	2	1
2	2	2	2	2	2	2	2	1
2	2	2	2	2	2	2	2	1
2	2	2	2	2	2	2	2	1
1	2	2	2	2	2	2	2	1
2	2	2	2	2	2	2	2	1
2	2	2	2	2	2	2	2	2
2	2	2	2	2	2	2	1	1
88	2	2	2	2	2	2	2	66
2	2	2	2	1	2	2	2	2
2	2	2	2	2	2	2	1	2
2	2	2	2	2	2	2	2	1
1	1	2	2	2	2	2	2	2
2	2	2	2	2	2	2	1	1
2	2	2	2	1	2	2	2	1
1	2	2	2	2	2	2	2	2
2	2	2	2	2	2	2	2	1
2	2	2	2	2	2	2	2	2
2	2	2	2	1	2	2	2	1
2	2	2	2	1	2	2	2	1
2	2	2	2	1	2	2	2	1
2	2	2	2	2	2	2	2	1

2	2	2	2	1	2	2	2	1
2	2	2	2	1	2	2	2	1
2	2	2	2	2	2	2	2	1
2	2	2	2	2	2	2	2	1
66	2	2	2	2	2	2	2	1
2	2	2	2	2	2	2	2	1
2	2	2	2	1	2	2	2	1
2	2	2	2	2	2	2	2	1
1	2	2	2	2	2	2	2	2
2	1	2	2	2	2	2	2	2
2	2	2	2	2	2	2	2	1
2	2	2	2	2	2	2	2	2
2	2	2	2	2	2	2	1	1
2	2	2	2	2	2	2	2	1
2	2	2	2	2	2	2	2	1
2	2	2	2	2	2	2	2	1
2	2	2	2	2	1	2	2	1
1	2	2	2	2	2	2	2	2
2	2	2	2	1	2	2	2	1
1	2	2	2	2	2	2	2	1
2	2	2	2	2	2	2	2	2
2	2	2	2	2	2	2	2	1
88	2	2	2	2	2	2	2	2
88	2	2	2	2	2	2	2	2
2	2	2	2	1	2	2	2	1
2	2	2	2	2	2	2	1	1
2	2	2	2	1	2	2	2	66
1	2	2	2	2	2	2	2	2
2	2	2	2	2	2	2	2	2
2	2	2	2	2	2	2	1	1
2	2	2	2	2	2	2	2	1
2	2	2	2	2	2	2	2	1
66	2	2	2	2	2	2	2	1
2	2	2	2	1	2	2	2	1
2	2	2	2	1	2	2	2	1
2	2	2	2	2	1	2	2	2
2	2	2	2	2	2	2	2	2
88	2	2	2	2	2	2	2	66
2	2	2	2	2	2	2	2	1
2	2	2	2	1	2	2	2	1
2	2	2	2	2	2	2	2	1
66	2	2	2	2	2	2	2	1
2	2	2	2	2	2	2	1	1
2	2	2	2	2	2	2	2	1
2	1	2	2	2	2	2	1	2
2	2	2	2	2	2	2	2	1
2	2	2	2	2	2	2	2	1
2	2	2	2	2	2	2	1	1
2	2	2	2	1	2	2	1	1
2	2	2	2	2	2	2	2	1

66	2	2	2	2	2	2	2	1
2	2	2	2	2	2	2	2	2
66	2	2	2	2	2	2	2	1
2	2	2	2	2	2	2	1	1
2	2	2	2	2	2	2	2	66
66	2	2	2	2	2	2	2	66
2	2	2	2	1	2	2	2	2
2	2	2	2	2	1	2	2	1
2	2	2	2	2	2	2	2	2
2	2	2	1	2	2	2	2	2
66	2	2	2	2	2	2	2	2
2	2	2	2	2	2	2	2	1
2	2	2	2	2	2	2	2	2
2	2	2	2	2	2	2	2	1
2	2	2	2	2	2	2	2	1
2	2	2	2	1	2	2	2	2
2	2	2	2	1	2	2	2	1
2	2	2	2	2	2	2	2	1
2	2	2	2	2	2	2	2	2
66	2	2	2	2	2	2	2	66
2	2	2	2	2	2	2	2	2
2	2	2	2	2	2	2	2	1
2	2	2	2	1	2	2	2	1
2	1	2	2	2	2	2	1	1
2	2	2	2	1	2	2	2	2
66	2	2	2	2	2	2	2	1
2	2	2	2	2	2	2	2	2
66	2	2	2	2	2	2	2	2
66	2	2	2	2	2	2	2	2
66	2	2	2	1	2	2	2	1
2	2	2	2	2	2	2	1	66
2	2	2	2	2	2	2	2	1
2	2	2	2	2	2	2	2	1
2	2	2	2	2	2	2	2	3
2	2	2	2	1	2	2	2	2
2	2	2	2	2	2	2	2	1
66	2	2	2	2	2	2	2	2
2	2	2	2	1	2	2	2	1
2	2	2	2	2	2	2	2	1
66	2	2	2	2	2	2	2	2
66	2	2	2	2	2	2	2	1
66	2	2	2	2	2	2	2	2
2	2	2	2	2	2	2	2	1
2	2	2	2	1	2	2	2	1
2	2	2	2	2	2	2	2	66
2	2	2	2	2	2	2	2	2
2	2	2	2	2	2	2	2	2
2	2	2	2	2	2	2	2	1
1	2	2	2	2	2	2	2	2
2	2	2	2	2	2	2	2	2

2	2	2	2	1	2	2	2	2
2	1	2	2	2	2	2	2	66
2	2	2	2	2	2	2	2	3
2	2	2	2	1	2	2	1	1
2	2	2	2	2	2	2	2	1
2	2	2	2	2	2	2	2	1
2	2	2	2	2	2	2	2	1
2	2	2	2	2	2	2	2	1
2	2	2	2	2	2	2	2	1
2	1	2	2	2	2	2	2	2
2	2	2	2	2	2	2	2	2
88	2	2	2	2	2	2	2	66
2	2	2	2	2	2	2	2	1
2	2	2	2	1	2	2	2	2
2	2	2	2	2	2	2	2	2
2	2	2	2	2	2	2	2	1
2	2	2	2	1	2	2	2	1
2	2	2	2	2	2	2	2	1
2	2	2	2	1	2	2	2	2
2	2	2	2	2	2	2	2	2
2	2	2	2	2	2	2	2	1
2	2	2	2	2	2	2	2	1
2	2	2	2	1	2	2	2	2
2	1	2	2	2	2	2	2	2
2	2	2	2	2	2	2	2	1
2	2	2	2	1	2	2	2	66
2	2	2	2	2	2	2	2	1
2	2	2	2	1	2	2	2	66
2	2	2	2	2	2	2	2	2
2	2	2	2	2	2	2	2	1
2	2	2	2	2	2	2	2	66
2	2	2	2	1	2	2	2	1
2	2	2	2	1	2	2	2	2
2	2	2	2	2	2	2	2	2
2	2	2	2	1	2	2	2	66
2	2	2	2	1	2	2	2	1
1	2	2	2	2	2	2	2	2
2	2	2	2	2	2	2	2	1
2	2	2	2	2	2	2	2	66
2	2	2	2	1	2	2	2	66
2	1	2	2	1	2	2	1	1
2	2	2	2	2	2	2	2	1
2	2	2	2	2	2	2	2	1
88	88	2	2	2	2	88	88	2
88	88	2	2	2	2	88	88	2
88	88	2	2	1	2	88	88	2
88	88	2	2	2	2	88	88	2
88	88	2	2	2	2	88	88	2
88	88	2	2	2	2	88	88	2
88	88	2	2	2	2	88	88	2

88	88	2	2	2	2	88	88	1
88	88	2	2	1	2	88	88	2
88	88	2	2	1	2	1	88	2
88	88	2	2	2	2	2	88	1
88	88	2	2	2	2	88	88	2
88	88	2	2	1	2	1	88	2
88	88	2	2	2	2	88	88	2
88	88	2	2	2	2	88	88	2
88	88	2	2	1	2	88	88	1
88	88	2	2	1	2	88	88	1
88	88	2	2	2	2	88	88	2
88	88	2	2	2	2	88	88	2
88	88	2	2	1	2	1	88	1
88	88	2	2	1	2	1	88	1
88	88	2	2	1	2	2	88	1
88	88	2	2	1	2	1	88	2
88	88	2	2	2	2	88	88	2
88	88	2	2	1	2	1	88	2
88	88	2	2	1	2	1	88	2
88	88	2	2	1	2	1	88	1
88	88	2	2	2	2	88	88	2
88	88	2	2	1	2	88	88	1
88	88	2	2	2	2	88	88	3
88	88	2	2	2	2	88	88	2
88	88	2	2	1	2	88	88	66
88	88	2	2	2	2	88	88	66
88	88	2	2	1	2	1	88	66
88	88	2	2	2	2	88	88	1
88	88	2	2	2	2	88	88	2
88	88	88	88	88	88	88	88	88
88	88	2	2	2	2	88	88	2
88	88	2	2	1	2	1	88	66
88	88	2	2	2	2	2	88	1
88	88	2	2	2	2	88	88	66
88	88	2	2	2	2	88	88	2
88	88	2	2	1	2	1	88	66
88	88	2	2	1	2	1	88	1
88	88	2	2	1	2	88	88	2
88	88	2	2	2	2	88	88	1
88	88	2	2	1	2	1	88	2
88	88	2	2	1	2	1	88	2
88	88	2	2	2	2	88	88	2
88	88	2	2	1	2	1	88	1
88	88	2	2	1	2	1	88	2
88	88	2	2	1	2	2	88	1
88	88	2	2	1	2	2	88	2
88	88	2	2	2	2	88	88	2
88	88	88	88	88	88	88	88	88
88	88	2	2	2	2	88	88	2
88	88	88	88	88	88	88	88	88

88	88	2	2	1	2	1	88	2
88	88	2	2	2	2	88	88	2
88	88	2	2	2	2	88	88	2
88	88	2	2	2	2	88	88	2
88	88	2	2	1	2	2	88	1
88	88	2	2	2	1	88	88	66
88	88	88	88	88	88	88	88	88
88	88	2	2	1	2	1	88	2
88	88	2	2	1	2	1	88	1
88	88	2	2	2	2	88	88	2
88	88	88	88	88	88	88	88	88
88	88	2	2	1	2	1	88	2
88	88	2	2	2	2	88	88	66
88	88	2	2	2	2	88	88	66
88	88	2	2	1	2	2	88	66
88	88	2	2	1	2	1	88	1
88	88	2	2	1	2	1	88	1
88	88	2	2	1	2	1	88	1
88	88	2	2	2	2	88	88	1
88	88	2	2	1	2	1	88	2
88	88	2	2	1	2	1	88	2
88	88	2	2	1	2	88	88	1
88	88	2	2	2	2	2	88	1
88	88	2	2	2	2	88	88	2
88	88	2	2	1	2	1	88	2
88	88	2	2	2	2	88	88	2
88	88	2	2	1	2	1	88	2
88	88	2	2	2	2	88	88	2
88	88	2	2	2	2	88	88	2
88	88	2	2	1	1	88	88	1
88	88	2	2	2	2	88	88	66
88	88	2	2	1	2	88	88	1
88	88	2	2	2	2	88	88	2

ALERTVERN	MOTIVOAL	VOLINFUNI	TP	TEMPOTP	SANGAUM	TONUSUTE	HPPCON	HPPALE
2	88	88	1	00:74	1	2	1	2
2	88	88	1	00:30	1	1	1	2
2	88	88	1	00:05	2	2	2	2
2	88	88	1	01:26	2	2	1	2
2	88	88	1	04:10	1	2	1	2
2	88	88	1	02:28	2	2	2	2
2	88	88	1	00:12	2	2	2	2
2	88	88	1	01:26	2	2	2	2
2	88	88	1	02:13	2	2	2	2
2	88	88	1	01:46	1	2	1	2
2	88	88	1	02:32	1	2	2	2
2	88	88	1	02:13	2	2	2	2
2	88	88	1	07:47	1	1	1	2
2	88	88	1	00:08	2	2	2	2
2	88	88	1	00:48	1	1	1	2
2	88	88	1	08:65	1	1	1	2
2	88	88	1	03:45	1	1	1	2
2	88	88	1	03:76	2	2	2	2
2	88	88	1	05:08	1	2	1	2
2	88	88	1	05:00	1	2	1	2
2	88	88	1	01:25	1	2	1	2
2	88	88	1	00:36	2	2	2	2
2	88	88	1	02:12	2	2	2	2
2	88	88	1	00:56	2	2	2	2
2	88	88	1	01:32	2	2	2	2
2	88	88	1	02:62	1	1	1	2
2	88	88	1	02:73	2	2	2	2
2	88	88	1	00:51	2	2	2	2
2	88	88	1	01:58	2	1	1	2
2	88	88	1	02:74	2	2	2	2
2	88	88	1	00:23	1	2	1	2
2	88	88	1	00:31	2	1	1	2
2	88	88	1	04:23	2	2	2	2
2	88	88	1	02:42	2	2	2	2
2	88	88	1	05:41	2	2	2	2
2	88	88	1	Período ex	2	2	2	2
2	88	88	1	01:31	1	1	1	2
2	88	88	1	03:66	2	2	1	2
2	88	88	1	01:66	2	2	2	2
2	88	88	1	01:31	2	2	2	2
2	88	88	1	05:04	2	2	2	2
2	88	88	1	03:50	2	2	2	2
2	88	88	1	00:27	2	2	2	2
2	88	88	1	04:12	2	2	2	2
2	88	88	1	02:54	1	2	1	2
2	88	88	1	02:70	2	2	2	2
2	88	88	1	03:46	2	2	2	2
2	88	88	1	01:24	2	2	2	2
2	88	88	1	02:43	1	2	1	2

2	88	88	1	01:69	1	2	1	2
2	88	88	1	03:69	2	2	2	2
2	88	88	1	01:58	1	2	2	2
2	88	88	1	05:00	2	2	2	2
2	88	88	1	00:26	1	2	2	2
2	88	88	1	02:55	2	2	2	2
2	88	88	1	03:55	2	2	2	2
2	88	88	1	03:03	2	2	2	2
2	88	88	1	00:30	1	2	2	2
2	88	88	1	01:50	1	2	2	2
2	88	88	1	04:63	1	2	1	2
2	88	88	1	00:38	1	2	1	2
2	88	88	1	07:21	2	2	2	2
2	88	88	1	01:29	1	2	1	2
2	88	88	1	01:36	2	2	2	2
2	88	88	1	04:54	2	2	2	2
2	88	88	1	02:45	2	1	1	2
2	88	88	1	01:29	2	2	2	2
2	88	88	1	05:55	1	2	1	2
2	88	88	1	00:35	2	2	2	2
2	88	88	1	05:47	2	1	1	2
2	88	88	1	00:10	2	2	2	2
2	88	88	1	Período ex	2	2	2	2
2	88	88	1	00:20	2	2	2	2
2	88	88	1	00:21	2	2	2	2
2	88	88	1	01:71	2	2	2	2
2	88	88	1	00:74	1	2	1	2
2	88	88	1	00:42	2	2	2	2
2	88	88	1	02:15	2	2	2	2
2	88	88	1	2:61	2	2	2	2
2	88	88	1	00:12	1	2	2	2
2	88	88	1	03:56	2	2	2	2
2	88	88	1	Período ex	2	2	2	2
2	88	88	1	01:47	1	2	1	2
2	88	88	1	01:37	2	2	2	2
2	88	88	1	01:82	1	2	1	2
2	88	88	1	00:24	1	2	1	2
2	88	88	1	00:30	2	2	2	2
2	88	88	1	01:34	2	2	2	2
2	88	88	1	01:62	1	2	1	2
2	88	88	1	05:72	1	1	1	2
2	88	88	1	00:30	1	1	1	2
2	88	88	1	04:20	2	2	2	2
2	88	88	1	07:20	2	2	2	2
2	88	88	1	07:52	2	2	2	2
2	88	88	1	03:10	1	2	2	2
2	88	88	1	05:23	2	2	2	2
2	88	88	1	02:65	2	2	2	2
2	88	88	1	05:89	1	1	1	2
2	88	88	1	02:40	2	1	1	2

2	88	88	1	00:07	2	2	2	2
2	88	88	1	04:74	1	1	1	2
2	88	88	1	00:24	2	2	2	2
2	88	88	1	04:84	2	2	2	2
2	88	88	1	03:86	2	2	2	2
2	88	88	1	00:10	2	1	1	2
2	88	88	1	00:04	2	2	2	2
2	88	88	1	07:86	2	2	2	2
2	88	88	1	00:18	2	2	2	2
2	88	88	1	02:28	2	1	1	2
2	88	88	1	00:40	2	2	2	2
2	88	88	1	04:80	2	2	2	2
2	88	88	1	00:23	1	1	1	2
2	88	88	1	01:48	2	2	2	2
2	88	88	1	01:33	2	1	1	2
2	88	88	1	00:72	1	2	1	2
2	88	88	1	03:30	2	2	2	2
2	88	88	1	01:37	2	2	2	2
2	88	88	1	01:01	2	2	2	2
2	88	88	1	00:19	2	2	2	2
2	88	88	1	01:91	2	2	2	2
2	88	88	1	01:47	1	1	1	2
2	88	88	1	00:23	1	2	1	2
2	88	88	1	02:51	2	2	2	2
2	88	88	1	02:19	2	2	2	2
2	88	88	1	00:10	2	2	2	2
2	88	88	1	07:29	2	2	2	2
2	88	88	1	00:40	2	2	2	2
2	88	88	1	00:25	2	2	2	2
2	88	88	1	00:80	1	2	1	2
2	88	88	1	09:26	1	1	1	2
2	88	88	1	03:62	2	2	2	2
2	88	88	1	00:46	2	2	2	2
2	88	88	1	01:18	2	2	2	2
2	88	88	1	03:85	2	2	2	2
2	88	88	1	05:38	2	2	2	2
2	88	88	1	00:05	2	2	2	2
2	88	88	1	01:42	1	2	1	2
2	88	88	1	02:59	2	2	2	2
2	88	88	1	01:35	2	2	2	2
2	88	88	1	00:26	2	2	2	2
2	88	88	1	00:79	2	2	1	2
2	88	88	1	05:58	2	2	1	2
2	88	88	1	01:47	2	2	2	2
2	88	88	1	01:34	1	2	1	2
2	88	88	1	01:16	1	2	1	2
2	88	88	1	00:16	2	2	2	2
2	88	88	1	01:46	2	2	1	2
2	88	88	1	00:35	2	2	2	2
2	88	88	1	00:36	2	2	2	2

2	88	88	1	00:36	2	2	2	2
2	88	88	1	00:63	2	1	1	2
2	88	88	1	00:20	2	2	2	2
2	88	88	1	04:54	2	2	2	2
2	88	88	1	05:51	2	2	1	2
2	88	88	1	00:62	2	2	2	2
2	88	88	1	01:84	2	2	2	2
2	88	88	1	00:29	2	2	2	2
2	88	88	1	00:48	2	2	2	2
2	88	88	1	03:12	1	2	1	2
2	88	88	1	01:60	2	2	2	2
2	88	88	1	Período ex	1	2	1	2
2	88	88	1	02:61	2	2	2	2
2	88	88	1	00:37	2	2	2	2
2	88	88	1	00:53	1	1	1	2
2	88	88	1	01:47	2	2	2	2
2	88	88	1	02:74	2	2	2	2
2	88	88	1	02:36	1	2	1	2
2	88	88	1	00:30	2	2	2	2
2	88	88	1	02:18	2	2	2	2
2	88	88	1	24:47	2	2	2	2
2	88	88	1	04:81	2	2	2	2
2	88	88	1	00:71	2	2	2	2
2	88	88	1	02:20	1	2	1	2
2	88	88	1	05:41	2	2	2	2
2	88	88	1	03:33	2	1	1	2
2	88	88	1	01:25	1	2	1	2
2	88	88	1	00:30	2	2	2	2
2	88	88	1	00:45	2	2	2	2
2	88	88	1	01:50	2	2	2	2
2	88	88	1	01:58	2	2	2	2
2	88	88	1	02:47	2	2	2	2
2	88	88	1	02:49	1	2	1	2
2	88	88	1	01:54	2	1	1	2
2	88	88	1	02:56	2	2	2	2
2	88	88	1	02:40	2	2	2	2
2	88	88	1	00:21	1	1	1	2
2	88	88	1	00:41	2	2	2	2
2	88	88	1	02:22	2	2	2	2
2	88	88	1	03:37	2	2	2	2
2	88	88	1	05:51	2	2	2	2
2	88	88	1	03:95	2	1	1	2
2	88	88	1	01:48	2	2	2	2
2	88	88	2	8888	2	2	2	2
2	88	88	2	8888	2	2	2	2
2	88	88	2	8888	2	2	2	2
2	88	88	2	8888	2	2	2	2
2	88	88	2	8888	2	2	2	2
2	88	88	2	8888	2	2	2	2
2	88	88	2	8888	2	2	2	2

2	88	88	2	8888	2	2	2	2
2	88	88	2	8888	2	2	2	2
2	88	88	1	04:43	1	2	1	2
2	88	88	1	6666	2	2	2	2
2	88	88	2	8888	2	2	2	2
2	88	88	1	05:57	2	2	2	2
2	88	88	2	8888	2	2	2	2
2	88	88	2	8888	1	2	1	2
2	88	88	2	8888	2	2	2	2
2	88	88	2	8888	2	2	2	2
2	88	88	2	8888	2	2	2	2
2	88	88	2	8888	2	2	2	2
2	88	88	1	04:32	2	2	2	2
2	88	88	1	04:20	2	2	2	2
2	88	88	1	08:47	2	2	2	2
2	88	88	1	04:29	2	2	2	2
2	88	88	2	8888	2	2	2	2
2	88	88	1	06:50	2	2	2	2
2	88	88	1	03:08	1	1	1	2
2	88	88	1	02:37	2	2	2	2
2	88	88	2	8888	2	2	2	2
2	88	88	2	8888	2	2	2	2
2	88	88	2	8888	2	2	2	2
2	88	88	2	8888	2	2	2	2
2	88	88	2	8888	2	1	1	2
2	88	88	2	8888	2	2	1	2
2	88	88	1	06:56	2	2	2	2
2	88	88	2	8888	1	2	1	2
2	88	88	2	8888	1	2	1	2
88	88	88	88	8888	88	88	88	88
2	88	88	2	8888	2	2	2	2
2	88	88	1	05:08	2	2	2	2
2	88	88	1	01:15	1	2	1	2
2	88	88	2	8888	2	2	2	2
2	88	88	2	8888	1	2	2	2
2	88	88	1	30:02	1	2	1	2
2	88	88	1	06:19	2	1	1	2
2	88	88	2	8888	2	2	2	2
2	88	88	2	8888	2	2	2	2
2	88	88	1	05:01	2	2	2	2
2	88	88	1	09:22	2	2	2	2
2	88	88	2	8888	1	1	1	2
2	88	88	1	10:47	2	2	2	2
2	88	88	1	03:24	2	2	2	2
2	88	88	1	09:29	2	2	2	2
2	88	88	1	04:23	1	2	1	2
2	88	88	2	8888	2	2	2	2
88	88	88	88	8888	88	88	88	88
2	88	88	2	8888	2	2	2	2
88	88	88	88	8888	88	88	88	88

2	88	88	1	02:29	2	2	2	2
2	88	88	2	8888	2	2	1	2
2	88	88	2	8888	2	2	2	2
2	88	88	2	8888	2	2	2	2
2	88	88	1	00:22	2	2	2	2
2	88	88	2	8888	2	2	2	2
88	88	88	88	8888	88	88	88	88
2	88	88	1	06:22	2	2	2	2
2	88	88	1	11:47	2	2	2	2
2	88	88	2	8888	2	2	2	2
88	88	88	88	8888	88	88	88	88
2	88	88	1	07:02	2	2	2	2
2	88	88	2	8888	2	2	2	2
2	88	88	2	8888	2	2	2	2
2	88	88	1	09:11	2	2	2	2
2	88	88	1	06:02	2	1	1	2
2	88	88	1	07:16	2	1	1	2
2	88	88	1	05:57	2	2	2	2
2	88	88	2	8888	2	2	1	2
2	88	88	1	06:26	2	2	2	2
2	88	88	1	08:29	2	2	2	2
2	88	88	2	8888	2	2	2	2
2	88	88	1	03:13	2	2	2	2
2	88	88	2	8888	2	2	1	2
2	88	88	1	09:36	2	2	1	2
2	88	88	2	8888	2	2	2	2
2	88	88	1	08:00	2	2	2	2
2	88	88	2	8888	2	2	2	2
2	88	88	2	8888	2	2	2	2
2	88	88	2	8888	2	2	2	2
2	88	88	2	8888	2	2	2	2
2	88	88	2	8888	2	2	2	2
2	88	88	2	8888	2	2	2	2

CONDUTHF	CODUTHPF	CONDUTHF	CONDUTHF	CONDUTHF	CONDUHPF	RETEPLA	CURE	CHAD
2	1	2	2	2	2	1	1	2
1	1	2	2	2	2	1	1	2
88	88	88	88	88	88	1	1	2
1	2	2	2	2	2	1	1	2
1	1	1	1	1	1	1	1	2
88	88	88	88	88	88	2	88	2
88	88	88	88	88	88	2	88	2
88	88	88	88	88	88	2	88	2
88	88	88	88	88	88	2	88	2
1	2	2	2	2	2	2	88	2
88	88	88	88	88	88	2	88	2
88	88	88	88	88	88	2	88	2
1	2	2	2	1	2	2	88	2
88	88	88	88	88	88	2	88	2
1	1	1	2	1	2	2	88	2
1	1	1	2	1	2	2	88	2
1	1	1	2	1	2	2	88	2
88	88	88	88	88	88	2	88	2
1	1	2	2	2	2	2	88	2
1	1	1	1	1	2	2	88	2
1	2	2	2	2	2	2	88	2
88	88	88	88	88	88	2	88	2
88	88	88	88	88	88	2	88	2
88	88	88	88	88	88	2	88	2
88	88	88	88	88	88	2	88	2
1	1	1	1	1	2	2	88	2
88	88	88	88	88	88	2	88	2
88	88	88	88	88	88	2	88	2
1	2	2	2	2	2	2	88	2
88	88	88	88	88	88	2	88	2
1	1	1	2	2	2	2	88	2
1	2	2	2	2	2	2	88	2
88	88	88	88	88	88	2	88	2
88	88	88	88	88	88	2	88	2
88	88	88	88	88	88	2	88	2
88	88	88	88	88	88	2	88	2
1	1	2	2	2	2	2	88	2
1	1	2	2	2	2	2	88	2
88	88	88	88	88	88	2	88	2
88	88	88	88	88	88	2	88	2
88	88	88	88	88	88	2	88	2
88	88	88	88	88	88	2	88	2
88	88	88	88	88	88	2	88	2
88	88	88	88	88	88	2	88	2
1	2	2	2	2	2	2	88	2
88	88	88	88	88	88	2	88	2
88	88	88	88	88	88	2	88	2
88	88	88	88	88	88	2	88	2
1	1	1	1	1	2	2	88	2

1	1	2	2	2	2	2	88	2
88	88	88	88	88	88	2	88	2
88	88	88	88	88	88	2	88	2
88	88	88	88	88	88	2	88	2
88	88	88	88	88	88	2	88	2
88	88	88	88	88	88	2	88	2
88	88	88	88	88	88	2	88	2
88	88	88	88	88	88	2	88	2
88	88	88	88	88	88	2	88	2
2	2	2	2	2	2	2	88	2
1	2	2	2	2	2	2	88	2
1	1	2	2	2	2	2	88	2
88	88	88	88	88	88	2	88	2
1	1	2	2	2	2	2	88	2
88	88	88	88	88	88	2	88	2
88	88	88	88	88	88	2	88	2
1	1	1	1	1	2	2	88	2
88	88	88	88	88	88	2	88	2
1	2	2	2	1	2	2	88	2
88	88	88	88	88	88	2	88	2
1	2	2	2	1	2	2	88	2
88	88	88	88	88	88	2	88	2
88	88	88	88	88	88	2	88	2
88	88	88	88	88	88	2	88	2
88	88	88	88	88	88	2	88	2
88	88	88	88	88	88	2	88	2
1	2	2	2	1	2	2	88	2
88	88	88	88	88	88	2	88	2
88	88	88	88	88	88	2	88	2
88	88	88	88	88	88	2	88	2
2	2	2	2	2	2	2	88	2
88	88	88	88	88	88	2	88	2
88	88	88	88	88	88	2	88	2
1	2	2	2	2	2	2	88	2
88	88	88	88	88	88	2	88	2
1	2	2	2	2	2	2	88	2
1	2	2	2	2	2	2	88	2
88	88	88	88	88	88	2	88	2
88	88	88	88	88	88	2	88	2
1	1	2	2	2	2	2	88	2
1	2	2	2	2	2	2	88	2
1	2	2	2	2	2	2	88	2
88	88	88	88	88	88	2	88	2
88	88	88	88	88	88	2	88	2
88	88	88	88	88	88	2	88	2
88	88	88	88	88	88	2	88	2
88	88	88	88	88	88	2	88	2
88	88	88	88	88	88	2	88	2
1	1	1	1	2	2	2	88	2
1	2	2	2	1	2	2	88	2

88	88	88	88	88	88	2	88	2
1	1	2	2	1	2	2	88	2
88	88	88	88	88	88	2	88	2
88	88	88	88	88	88	2	88	2
88	88	88	88	88	88	2	88	2
1	2	2	2	2	2	2	88	2
88	88	88	88	88	88	2	88	2
88	88	88	88	88	88	2	88	2
88	88	88	88	88	88	2	88	2
1	2	2	2	2	2	2	88	2
88	88	88	88	88	88	2	88	2
88	88	88	88	88	88	2	88	2
1	1	1	1	1	2	2	88	2
88	88	88	88	88	88	2	88	2
1	1	1	2	2	2	2	88	2
1	2	2	2	2	2	2	88	2
88	88	88	88	88	88	2	88	2
88	88	88	88	88	88	2	88	2
88	88	88	88	88	88	2	88	2
88	88	88	88	88	88	2	88	2
88	88	88	88	88	88	2	88	2
1	1	2	2	2	2	2	88	2
1	2	2	2	1	2	2	88	2
88	88	88	88	88	88	2	88	2
88	88	88	88	88	88	2	88	2
88	88	88	88	88	88	2	88	2
88	88	88	88	88	88	2	88	2
88	88	88	88	88	88	2	88	2
88	88	88	88	88	88	2	88	2
1	2	2	2	2	2	2	88	2
1	1	1	1	1	2	2	88	2
88	88	88	88	88	88	2	88	2
88	88	88	88	88	88	2	88	2
88	88	88	88	88	88	2	88	2
88	88	88	88	88	88	2	88	2
88	88	88	88	88	88	2	88	2
88	88	88	88	88	88	2	88	2
1	1	2	2	2	2	2	88	2
88	88	88	88	88	88	2	88	2
88	88	88	88	88	88	2	88	2
88	88	88	88	88	88	2	88	2
1	1	2	2	2	2	2	88	2
1	1	2	2	2	2	2	88	2
88	88	88	88	88	88	2	88	2
1	2	2	2	2	2	2	88	2
1	1	2	2	2	2	2	88	2
88	88	88	88	88	88	2	88	2
1	1	2	2	2	2	2	88	2
88	88	88	88	88	88	2	88	2
88	88	88	88	88	88	2	88	2

88	88	88	88	88	88	2	88	2
1	2	2	2	2	2	2	88	2
88	88	88	88	88	88	2	88	2
88	88	88	88	88	88	2	88	2
1	2	2	2	1	2	2	88	2
88	88	88	88	88	88	2	88	2
88	88	88	88	88	88	2	88	2
88	88	88	88	88	88	2	88	2
88	88	88	88	88	88	2	88	2
1	2	2	2	2	2	2	88	2
88	88	88	88	88	88	2	88	2
1	2	2	2	2	2	2	88	2
88	88	88	88	88	88	2	88	2
88	88	88	88	88	88	2	88	2
1	1	2	1	1	2	2	88	2
88	88	88	88	88	88	2	88	2
88	88	88	88	88	88	2	88	2
1	1	1	1	1	2	2	88	2
88	88	88	88	88	88	2	88	2
88	88	88	88	88	88	2	88	2
88	88	88	88	88	88	2	88	2
88	88	88	88	88	88	2	88	2
88	88	88	88	88	88	2	88	2
1	1	2	2	1	2	2	88	2
88	88	88	88	88	88	2	88	2
1	2	2	2	2	2	2	88	2
1	1	2	2	2	2	2	88	2
88	88	88	88	88	88	2	88	2
88	88	88	88	88	88	2	88	2
88	88	88	88	88	88	2	88	2
88	88	88	88	88	88	2	88	2
88	88	88	88	88	88	2	88	2
1	1	2	2	1	2	2	88	2
1	1	1	2	2	2	2	88	2
88	88	88	88	88	88	2	88	2
88	88	88	88	88	88	2	88	2
1	2	2	2	2	2	2	88	2
88	88	88	88	88	88	2	88	2
88	88	88	88	88	88	2	88	2
88	88	88	88	88	88	2	88	2
88	88	88	88	88	88	2	88	2
1	2	1	2	2	2	2	88	2
88	88	88	88	88	88	2	88	2
88	88	88	88	88	88	88	88	2
88	88	88	88	88	88	88	88	2
88	88	88	88	88	88	88	88	2
88	88	88	88	88	88	88	88	2
88	88	88	88	88	88	88	88	2
88	88	88	88	88	88	88	88	2
88	88	88	88	88	88	88	88	2

88	88	88	88	88	88	88	88	2
88	88	88	88	88	88	88	88	2
1	1	1	1	1	2	88	88	2
88	88	88	88	88	88	88	88	2
88	88	88	88	88	88	88	88	2
88	88	88	88	88	88	88	88	2
88	88	88	88	88	88	88	88	2
1	2	2	2	2	2	88	88	2
88	88	88	88	88	88	88	88	2
88	88	88	88	88	88	88	88	2
88	88	88	88	88	88	88	88	2
88	88	88	88	88	88	88	88	2
88	88	88	88	88	88	88	88	2
88	88	88	88	88	88	88	88	2
88	88	88	88	88	88	88	88	2
88	88	88	88	88	88	88	88	2
88	88	88	88	88	88	88	88	2
88	88	88	88	88	88	88	88	2
1	1	1	2	2	1	88	88	2
88	88	88	88	88	88	88	88	2
88	88	88	88	88	88	88	88	2
88	88	88	88	88	88	88	88	2
88	88	88	88	88	88	88	88	2
88	88	88	88	88	88	88	88	2
1	2	1	2	2	2	88	88	2
1	1	1	1	2	2	88	88	2
88	88	88	88	88	88	88	88	2
1	2	2	2	2	2	88	88	2
1	1	1	2	2	2	88	88	2
88	88	88	88	88	88	88	88	88
88	88	88	88	88	88	88	88	2
88	88	88	88	88	88	88	88	2
1	2	2	2	2	2	88	88	2
88	88	88	88	88	88	88	88	2
88	88	88	88	88	88	88	88	2
1	2	2	2	2	2	88	88	1
1	1	1	1	2	2	88	88	1
88	88	88	88	88	88	88	88	2
88	88	88	88	88	88	88	88	2
88	88	88	88	88	88	88	88	2
88	88	88	88	88	88	88	88	2
1	2	2	2	2	2	88	88	2
88	88	88	88	88	88	88	88	2
88	88	88	88	88	88	88	88	2
88	88	88	88	88	88	88	88	2
1	1	2	2	2	2	88	88	2
88	88	88	88	88	88	88	88	2
88	88	88	88	88	88	88	88	88
88	88	88	88	88	88	88	88	2
88	88	88	88	88	88	88	88	88

88	88	88	88	88	88	88	88	2
1	1	2	1	2	2	88	88	2
88	88	88	88	88	88	88	88	2
88	88	88	88	88	88	88	88	2
88	88	88	88	88	88	88	88	2
88	88	88	88	88	88	88	88	2
88	88	88	88	88	88	88	88	88
88	88	88	88	88	88	88	88	2
88	88	88	88	88	88	88	88	2
88	88	88	88	88	88	88	88	2
88	88	88	88	88	88	88	88	88
88	88	88	88	88	88	88	88	2
88	88	88	88	88	88	88	88	2
88	88	88	88	88	88	88	88	2
88	88	88	88	88	88	88	88	2
1	1	2	1	2	2	88	88	2
1	1	1	2	2	2	88	88	2
88	88	88	88	88	88	88	88	2
1	2	1	2	2	2	88	88	2
88	88	88	88	88	88	88	88	2
88	88	88	88	88	88	88	88	2
88	88	88	88	88	88	88	88	2
88	88	88	88	88	88	88	88	2
1	1	2	2	2	2	88	88	2
1	1	2	2	2	2	88	88	2
88	88	88	88	88	88	88	88	2
88	88	88	88	88	88	88	88	2
88	88	88	88	88	88	88	88	2
88	88	88	88	88	88	88	88	2
88	88	88	88	88	88	88	88	2
88	88	88	88	88	88	88	88	2
88	88	88	88	88	88	88	88	2
88	88	88	88	88	88	88	88	2
